# Reconstitution of a functional human thymus by postnatal stromal progenitor cells and natural whole-organ scaffolds

**DOI:** 10.1038/s41467-020-20082-7

**Published:** 2020-12-11

**Authors:** Sara Campinoti, Asllan Gjinovci, Roberta Ragazzini, Luca Zanieri, Linda Ariza-McNaughton, Marco Catucci, Stefan Boeing, Jong-Eun Park, John C. Hutchinson, Miguel Muñoz-Ruiz, Pierluigi G. Manti, Gianluca Vozza, Carlo E. Villa, Demetra-Ellie Phylactopoulos, Constance Maurer, Giuseppe Testa, Hans J. Stauss, Sarah A. Teichmann, Neil J. Sebire, Adrian C. Hayday, Dominique Bonnet, Paola Bonfanti

**Affiliations:** 1 Epithelial Stem Cell Biology & Regenerative Medicine laboratory, The Francis Crick Institute, 1 Midland Road, London, NW1 1AT UK; 2 UCL Great Ormond Street Institute of Child Health, 30 Guilford Street, London, WC1N 1EH UK; 3 Institute of Immunity & Transplantation, Division of Infection & Immunity, UCL, Royal Free Hospital, London, NW3 2PF UK; 4 Haematopoietic Stem Cell laboratory, The Francis Crick Institute, 1 Midland Road, London, NW1 1AT UK; 5 Bioinformatics Laboratory, The Francis Crick Institute, 1 Midland Road, London, NW1 1AT UK; 6 Wellcome Sanger Institute, Wellcome Genome Campus, Hinxton, Cambridge, CB10 1SA UK; 7 Department of Histopathology, Great Ormond Street Hospital for Children NHS Foundation Trust, London, WC1N 1EH UK; 8 Immunosurveillance laboratory, The Francis Crick Institute, 1 Midland Road, London, NW1 1AT UK; 9 Department of Experimental Oncology, IEO, European Institute of Oncology, IRCCS, Milan, Italy; 10 Department of Oncology and Hemato-Oncology, University of Milan, Milan, Italy; 11 Peter Gorer Department of Immunobiology, School of Immunology & Microbial Sciences, King’s College London, London, UK; 12 Present Address: Division of Immunology, Transplantation and Infectious Diseases, San Raffaele Scientific Institute, DIBIT 20132, Milan, Italy; 13 Present Address: Graduate School of Medical Science and Engineering, Korea Advanced Institute of Science and Technology (KAIST), Daejeon, 34141 Republic of Korea

**Keywords:** Regenerative medicine, Cell biology, Thymus, Regeneration

## Abstract

The thymus is a primary lymphoid organ, essential for T cell maturation and selection. There has been long-standing interest in processes underpinning thymus generation and the potential to manipulate it clinically, because alterations of thymus development or function can result in severe immunodeficiency and autoimmunity. Here, we identify epithelial-mesenchymal hybrid cells, capable of long-term expansion in vitro, and able to reconstitute an anatomic phenocopy of the native thymus, when combined with thymic interstitial cells and a natural decellularised extracellular matrix (ECM) obtained by whole thymus perfusion. This anatomical human thymus reconstruction is functional, as judged by its capacity to support mature T cell development in vivo after transplantation into humanised immunodeficient mice. These findings establish a basis for dissecting the cellular and molecular crosstalk between stroma, ECM and thymocytes, and offer practical prospects for treating congenital and acquired immunological diseases.

## Abstract (web summary)

The thymus is essential for T cell maturation and selection, and thymic defects result in severe immune problems. Here the authors identify a thymus cell population that is expandable in vitro, and can repopulate natural thymic matrix to generate tissue that supports mature T cell development in vitro and in vivo.

## Introduction

The thymus is a primary lymphoid organ where haematopoietic progenitors are instructed to become functional T cells. It undergoes postnatal involution, but remains in place throughout life^1,2^. The epithelial component of the thymus stroma derives from the endoderm, whereas mesenchymal stroma derives from the neural crest and in part from incoming vessels^3,4^. Reflecting their fundamental role in immune regulation, many studies have provided insight into the origins and phenotypic complexity of thymic epithelial cells (TEC)^5^ and the molecules that mediate their activities^6–10^, including AIRE and MHC Class II (MHCII) that are required for selecting T cells of diverse, non-autoaggressive specificities^5,11^.

A transcriptomic analysis describing human thymic complexity at the single-cell level was recently reported^12^. A core, long-established discriminator of TEC is their assignment as either cortical (cTEC) or medullary (mTEC), reflecting the anatomical regions to which the differentiated TEC contribute. However, while defined TEC and other stromal cell types have been studied in considerable detail, there is only incomplete molecular and cellular detail about how TEC and other cells collectively contribute to the complex three-dimensional (3D) organisation of the human thymus.

Since the thymus controls the development of both immune competence and tolerance, its functional dissection and subsequent reconstruction with desired cell populations might provide powerful tools applicable to many medical conditions, including primary or acquired immune deficiencies. Indeed, such is the clinical unmet need that cultivated thymus slices are currently used for transplantation into athymic patients. Moreover, thymic therapeutic strategies might likewise be applicable to organ transplantation in which tolerance is a key obstacle to long-term graft acceptance.

This notwithstanding, attempts to rebuild a fully functional thymus have so far met with limited success^13–15^, possibly because of the organ’s inherent complexity. Moreover, there remains some uncertainty over the nature of postnatal epithelial stem/progenitor and mesenchymal cells including the capacity for extensive expansion ex vivo that would seem crucial to this aim^16–18^. In this regard, we previously identified clonogenic multipotent stem cells in the rodent thymus able of contributing to the skin and thymic microenvironment after extensive ex vivo expansion^19^.

Our current study demonstrates that long-term expanding epithelial cells are present in the human thymus, and display an hitherto unprecedented hybrid epithelial–mesenchymal phenotype. These clonogenic cells could repopulate whole-organ scaffolds, phenocopying the unique 3D epithelial network of the thymus. Such reconstituted scaffolds established a functional microenvironment supporting human T cell development from lymphoid progenitors in vitro and haematopoietic stem cells (HSC) in vivo. Thus, by overcoming obstacles to constructing a functional thymus with only expanded, postnatal donor stromal cells, our findings offer practical prospects for treating immune disorders including congenital athymia for which current therapies are limited^20^.

## Results

### Long-term in vitro expansion identifies clonogenic TEC

**Fig. 1:**
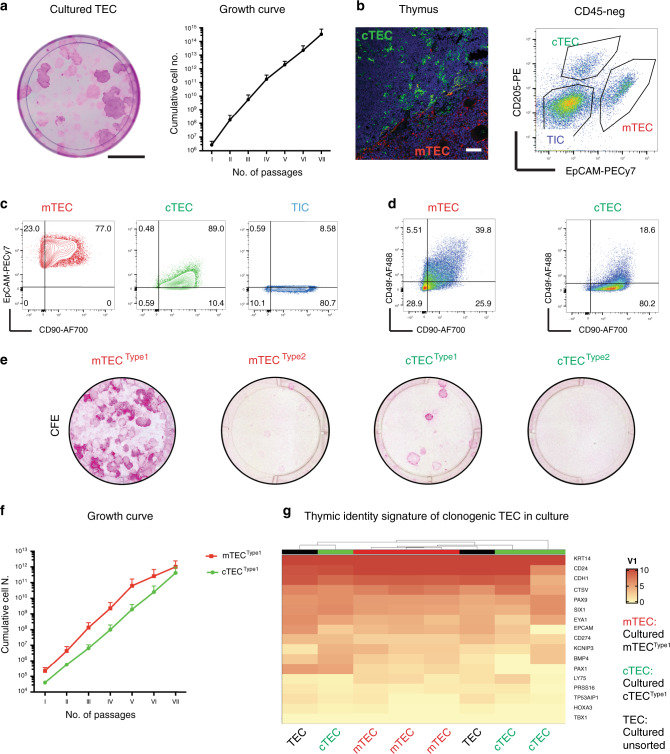
Prospective isolation of thymic epithelial (TEC) and interstitial cells (TIC). **a** Left panel: Rhodamine-B staining of TEC: 500 cultured TEC were seeded in a dish for colony-forming efficiency (CFE); the dish was stained after 12 days of culture. TEC gave rise to colonies of variable sizes that stained differently with Rhodamine. Scale bar, 2 cm. Right panel: growth curve over serial weekly passaging of TEC (*X* axis, number of weekly passages; *Y* axis, cumulative cell number); significance, mean ± SD (*n* = 3; donor age from 2 months to 4 years). **b** Left panel: immunofluorescence labelling of thymic epithelial cells in human post-natal thymus where anti-CD205 antibody (green) marked cortical TEC (cTEC) and anti-CK5-14 (red) marked medullary TEC (mTEC). Scale bar, 50 μm. Right panel: FACS plot visualising cTEC (EpCAM^low^CD205^+^, 4.87%), mTEC (EpCAM^high^CD205^−^, 11.1%) and TIC (EpCAM^−^CD205^−^, 61.5%) in the CD45-negative population enriched for sorting (*n* = 18; age from 3 days to 6 years). Representative analysis, thymus donor age 4 months (live cells = 51600). **c** FACS plots showing CD90 expression in enriched EpCAM^high^ mTEC (red), EpCAM^low^ cTEC (green) and EpCAM^−^ TIC (blue) respectively. Gating was decided on negative unstained control value for each channel. Same thymus as in 1b. **d** Representative FACS analysis for CD49f and CD90 demonstrating CD49f^+^ (Type1) and CD49f^−^ (Type2) populations. Same thymus as in 1b and 1c. **e** Rhodamine-stained indicator dishes of sorted cTEC and mTEC subtypes (4000 events/dish) cultivated for 12 days, colony-forming efficiency (CFE). mTEC^Type1^ is the most clonogenic population (2–4%) and cTEC^Type1^ (1–2%) is the clonogenic population of the cortex (*n* = 4). **f** Growth curve over serial weekly passaging of sorted mTEC^Type1^ and cTEC^Type1^ (*X* axis, number of weekly passages; *Y* axis, cumulative cell number; *n* = 4 independent cultures; data are presented as mean value ± SE; donor age from 2 months to 4 years old). **g** Gene expression signature of cultivated clonogenic TEC (unsorted, black), medullary (Type1 mTEC, red) and cortical TEC (Type1 cTEC, green). Heatmap and hierarchical clustering of samples according to expression of thymic identity genes (*SIX1, EYA1, PAX1* and *PAX9*), markers of thymic cortex (*CTSV, PRSS16* and *LY75*) and medulla (*CD24* and *CDH1*).

As a first experimental goal we attempted to isolate from human thymus stromal cells with clonogenic potential. Human thymi were obtained from 33 patients ranging in age from 3 days to 11 years who underwent open-chest cardiac surgery. Thymus tissues were enzymatically dissociated to single-cell suspension and plated over a lethally irradiated feeder layer for the derivation of thymic epithelial cells (TEC). All thymi, independent of donor age, contained clonogenic TEC generating colonies that could be expanded extensively upon weekly passage (Fig. 1a).

Subsequently, we aimed at defining if these clonogenic TEC derive from the cortex and/or medulla. To this end, we adopted a cell-sorting strategy to dissect and prospectively isolate thymic stromal compartments including the epithelium (cortex and medulla TEC) and the interstitium (TIC). After a few cycles of enrichment, the CD45^−^ stromal population went from 0.02–2% (freshly dissociated tissue) to ~40–80% (Supplementary Fig. 1a), allowing subsequent sorting on the basis of additional surface markers. We used EpCAM and CD205 expression levels^21,22^ to separate EpCAM^high^CD205^−^ medullary TEC (mTEC) from EpCAM^low^CD205^+^ cortical TEC (cTEC; Fig. 1b). Unexpectedly, the mesenchymal marker CD90 (THY1) was also expressed by a large proportion (>60%) of mTEC and approximately all cTEC, as well as by TIC (Fig. 1c). CD49f, the α6-integrin which contributes to hemidesmosomes and is expressed by cells at the basal layer of stratified epithelia^23,24^, was differentially expressed in both mTEC and cTEC populations (Fig. 1d), providing a basis to sort for CD49f^+^ mTEC or cTEC (“Type1”), and CD49f^−^ mTEC or cTEC (“Type2”) subpopulations.

In support of our sorting strategy, we employed RT-qPCR to assess expression of established functional markers of freshly isolated medullary (e.g. *AIRE1*) and cortical (e.g. *β−5T, CD205*) epithelial cells, respectively (Supplementary Fig. 1b). We noted differential expression in cortical *versus* medullary cells of cytokines and key transcription factors for thymus specification and/or thymopoiesis^25^ such as *FOXN1* and *PAX1* whereas Vimentin (*VIM*) was comparably expressed by all sorted populations examined (Supplementary Fig. 1c).

To assess the colony-forming potential of freshly isolated Type1 and Type2 human cTEC and mTEC, we plated them over lethally irradiated feeders, as described above. The colony-forming efficiency (CFE) assay consistently revealed that the highest clonogenicity resided in Type1 mTEC (CFE 2–4%) and Type1 cTEC (1–2%), while Type2 mTEC had a very low CFE (0.1–0.2%) and Type2 cTEC did not give rise to any expanding colonies (Fig. 1e). Entirely consistent with their greater clonogenicity, Type1 mTEC and Type1 cTEC were the only epithelial subpopulations that could be expanded over serial passages. Type1 cTEC, although being less abundant than their medulla counterparts, robustly expanded in culture and could reach comparable numbers to Type1 mTEC (Fig. 1f and Supplementary Fig. 1d).

In order to define the transcriptional profile of thymic clonogenic cells, we performed genome-wide RNA sequencing of cultivated Type1 mTEC, Type1 cTEC and also of TEC derived in culture without any sorting strategy. Expression of genes such as *SIX1, EYA1, HOXA3, PAX1* and *PAX9* detected by RNA sequencing of cultured TEC, confirmed that clonogenic TEC maintained thymic identity independently of their compartment of origin (cortical or medullary). Both cortical (*CSTV, KNICP3, CD274*) and medullary (*CDH1, EpCAM, CD24*) genes were retained by cultured cells (Fig. 1g). When analysed by RT-qPCR for transcription factors with documented importance for thymic epithelial cells, both mTEC and cTEC clonogenic cells expressed comparable levels of RNAs encoding *TP63*, *FOXN1* and signalling molecules such as SCF (Supplementary Fig. 1e). Indeed, mTEC and cTEC showed only 11 differentially expressed genes out of 14,347 (0.08%) thus confirming that in culture they display a common phenotype (Supplementary Fig. 2a, b). Thus, in subsequent experiments, we refer to “clonogenic TEC” whether or not they originate from medullary or cortical zones.

### Single-cell RNA sequencing defines cell clusters common to medullary and cortical populations in vivo

With the intention of using clonogenic cells expanded in vitro for thymus reconstitution, we first wanted to contextualise the cells’ profiles relative to freshly isolated mTEC and cTEC, directly ex vivo. Thus, freshly isolated subpopulations of mTEC and cTEC, Type1 and Type2, were subjected to single-cell RNA sequencing (scRNA-seq), placing them into the broader context of other murine and human scRNA-seq studies^12,26–30^. This combined approach offered the opportunity for high resolution profiling of freshly isolated thymic epithelial cells enriched for clonogenic cells.

**Fig. 2:**
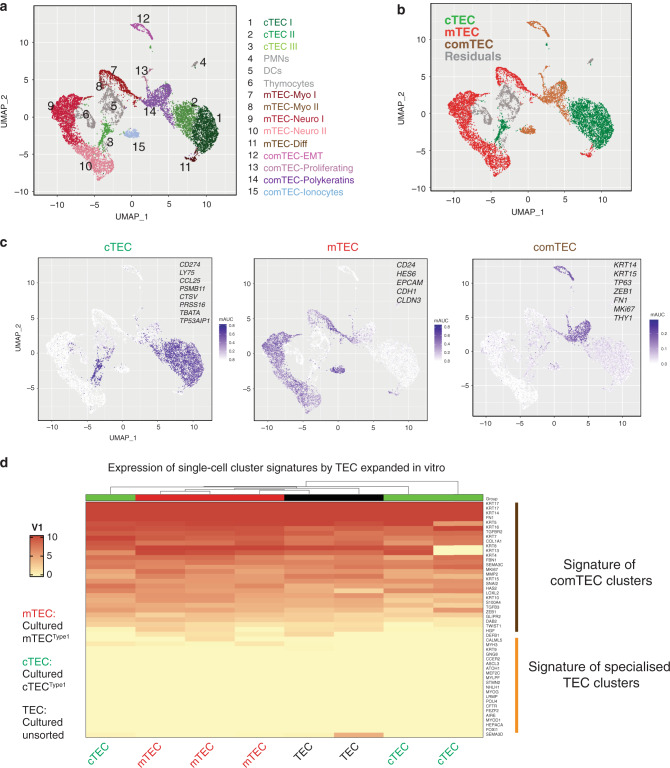
Single-cell RNA sequencing of freshly isolated TEC defines common cell clusters to mTEC and cTEC that are maintained in vitro. **a** UMAP visualisation of the cellular composition of the human post-natal thymus by clusters indicated by colours and numbers from 1 to 15 describing the heterogeneity of cTEC (1–3) and mTEC (7-11). Clusters 12–15 were common to cortex and medulla (comTEC); clusters 4–6 described residual immune cells (PMNs, DCs and thymocytes). **b** UMAP representation of the same thymic cells indicating main subgroups (cTEC, cortical epithelial cells, in green; mTEC, medullary epithelial cells, in red; TEC cells with common features, in brown and residuals of immune cells in grey). **c** Area under the curve (AUC) summary intensity plots for the gene signatures of cTEC (*CCL25, CD274, CFC1, CTSV, FOXN1, KCNIP3, LY75, PRSS16, PSMB11, SCX, SLC46A2, TBATA* and *TP53AIP1*), mTEC (*CD24, CDH1, CLDN3, EPCAM* and *HES6*) and comTEC (*CCL19, CFTR, COL1A1, DAB2, FN1, GLIPR2, HAS2, HGF, KRT14, KRT15, LOXL2, MKI67, MMP2, S100A4, SNAI1, SNAI2, TFCP2L1, TGFB3, TGFBR2, THY1, TP63, TWIST1* and *ZEB1*) clusters showing expression level for lineage specific genes in a purple scale. Single-cell expression values are represented as calculated median intensity of area under the curve. **d** Heatmap and hierarchical clustering of cultured cTEC^Type1^ (green, *n* = 3), mTEC^Type1^ (red, *n* = 3) and cultured unsorted TEC (black, *n* = 2) according to main genes clusters defined in single-cell RNA sequencing. Genes list is displayed on the right and includes comTEC signatures (epithelial-to-mesenchymal transition (EMT), polykeratins and proliferative - clusters 12-13-14) and specialised cell clusters: Myo (clusters 7-8), Neuro (clusters 9–10), Ionocytes (cluster 15) and differentiated mTEC (cluster 11).

When the mTEC and cTEC subpopulations were presented in a single UMAP (Fig. 2a), we were able to annotate main cell subgroups among sorted epithelial cells, defining 15 cell clusters distinguishable on the basis of shared common features: cTEC grouped within three main clusters, while mTEC grouped within five, in addition to which we identified four TEC clusters common to both cortex and medulla (“comTEC”; Fig. 2b). Note that the remaining three clusters (residual cells) represented polymorphonuclear, dendritic cell and thymocyte contaminants from the single-cell sorting (Fig. 2b). These main cell subgroups were validated by specific gene signatures of cTEC, mTEC or comTEC visualised in category view feature plots (Fig. 2c). Examples of single gene patterns of expression for each category (cTEC, mTEC and comTEC) are shown in Supplementary Fig. 2c–e for all the sorted Type1 and Type2 TEC.

Provocatively, the comTEC clusters included cells characterised by an epithelial–mesenchymal transition (EMT) signature (cluster 12), proliferation markers (cluster 13) and poly-keratin-expressing cells (cluster 14), while cluster 15 displayed a distinctive signature enriched in ion transporters^31^ (Fig. 2c). When we compared the transcriptional signatures of comTEC and of specific mTEC and cTEC clusters to the transcriptional profile of clonogenic TEC in vitro, it was evident that the latter expressed transcriptional signatures present in comTEC (proliferation, EMT and polykeratins) but not in other clusters of freshly isolated mTEC or cTEC (Fig. 2d).

### TEC co-express epithelial and mesenchymal markers but are distinct from thymic interstitial cells

**Fig. 3:**
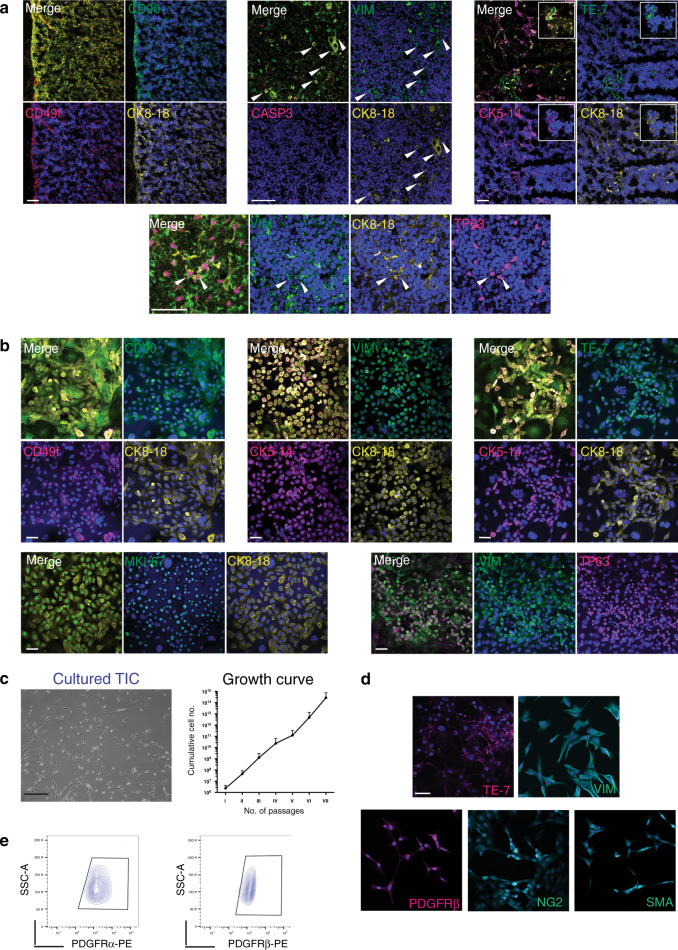
Phenotypic analysis of in vitro expanding thymic stromal cells. **a** Immunofluorescence labelling of thymic epithelial cells in human post-natal thymus where CK8-18 antibody (yellow), CK5-14 or CD49f (magenta) marked TEC. Tissues contains healthy cells as shown by absence of CASP3 (top mid panel). Co-staining with CD90 (top left panel), vimentin (VIM, top mid panel) or TE7 (top right panel) in green shows epithelial cells with mesenchymal features. Bottom panel shows co-expression of VIM (green), CK8-18 (yellow) and transcription factor TP63 (magenta). Arrow and inserts (white) highlight individual cells with hybrid phenotype. Nuclei are stained with DAPI. Scale bar, 50 μm (*n* = 4 thymi). **b** Immunofluorescence labelling of epithelial and mesenchymal markers on expanding thymic epithelial cells colonies expressing CD90 (left panels), VIM (middle panels) and TE7 (right panels) in green. Most of TEC in culture co-express cytokeratin CK5-14 (magenta) and CK8-18 (yellow), CD49f (magenta) together with mesenchymal markers. Bottom panels: left, TEC express proliferation marker MKI67 (green) and right panels: TEC co-express transcription factor TP63 (magenta) with VIM (green). Nuclei are stained with DAPI (*n* = 4 independent cultures). Scale bar, 50 μm. **c** Left panel: phase contrast image of thymic interstitial cells (TIC) expanded after sorting. Scale bar, 200 μm. Right panel: growth curve shows TIC expansion over several weekly passages (*n* = 4 independent cultures, data are presented as mean value ± S.D.). **d** Immunofluorescence of cultivated TIC demonstrates protein expression for TE7 and vimentin (VIM), as well as PDGFRβ, NG2 and Smooth Muscle Actin (αSMA), *n* = 3 independent cultures. Scale bar, 40 μm. **e**, Representative FACS analysis of expanded TIC for mesoderm markers PDGFRα (PE), PDGFRβ (PE), *n* = 5 independent cultures.

Based on the striking co-expression of CD90 (THY1) with EpCAM observed by FACS (Fig. 1c) and also by the detection of an EMT signature in the comTEC cluster of freshly isolated TEC (Supplementary 2f), we further investigated the possibility that human thymic epithelial cells might be characterised by a distinctive epithelial–mesenchymal hybrid phenotype. To this end, we performed immunohistochemistry analysis on postnatal thymi of our donors and clearly detected cells consistently co-expressing cytokeratins (CK) with mesenchymal markers such as CD90, vimentin (VIM) and to a less extent, the mesoderm marker TE7 (Fig. 3a), thus confirming at the protein level a hybrid phenotype expressed within healthy thymus tissue.

In culture, clonogenic TEC were CD49f^+^, in active cell cycle (MKI67^+^) and double positive for CK5-14 and CK8-18. In contrast to all epithelia described in culture so far, but in line with what reported above, clonogenic TEC stably co-expressed mesenchymal markers such as CD90, VIM and TE7 (Fig. 3b). In addition, some TEC expressing TP63, a transcription factor of the basal layer of stratified epithelia and a master regulator of thymic epithelial cells^32^, were also positive for VIM in vitro and in vivo (Fig. 3a, b). Genome-wide transcriptomic analysis of cultured TEC confirmed the expression in vitro of the EMT signature defined by scRNA sequencing (Supplementary Fig. 2g). Strikingly, expression of mesenchymal markers also correlated with high motility and migration that are typical of mesenchymal cells, as demonstrated by live-imaging of a TEC colony over 4 days in culture (Supplementary Movie 1). Thus, the hybrid epithelial–mesenchymal phenotype of epithelial cells appeared to be a highly distinctive feature of the thymus in vivo and ex vivo.

In addition to TEC, we also expanded CD90^+^EpCAM^−^ interstitial cells (Fig. 1c [TIC]). These cells did not grow under culture conditions designed for epithelial cells, but proliferated extensively for many passages in mesoderm culture conditions^33^, thus allowing us to obtain billions of cells in only a few weeks (Fig. 3c). Immunofluorescence showed expression of mesenchymal markers such as TE7, VIM, PDGFRβ, Chondroitin Sulfate Proteoglycan 4 (NG2) and Smooth Muscle Actin (αSMA; Fig. 3d). FACS analysis revealed that these TIC consistently expressed PDGFRα, PDGFRβ, CD90, CD146, and, to variable extents, NG2 and alkaline phosphatase (ALP), consistent with a perivascular phenotype^34^ (Fig. 3e and Supplementary Fig. 3a). FACS analysis of expanding TIC showed no presence of either epithelial (CD49f^+^) or immune (hCD45^+^) cells (Supplementary Fig. 3b).

Finally, we compared the transcriptional profile of the cultured epithelial clonogenic cells and thymic interstitial cells from paediatric samples of different age by genome-wide RNA sequencing. As expected, cultivated TEC appeared clearly distinct from the TIC as illustrated by the principal component analysis (PCA1; Supplementary Fig. 3c). Nevertheless, the data confirmed that the two cell types also share an exquisite similarity in the expression of genes related to EMT, including highly expressed *COL1A1*, *MMP2*, *TGFBR2* and *FN1* (Supplementary Fig. 3d).

### Natural thymic scaffolds

Given the capacity of TEC and TIC to expand substantially under the culture conditions employed, it became possible to obtain billions of TEC and TIC within a relatively short time-period, establishing them as putative stem/progenitor cells with clinical applicability. In order to assess their capacity to functionally differentiate we developed a unique approach to obtain thymic extracellular matrix (ECM) by whole-organ decellularisation that would provide a 3D structure for seeding thymic stroma and guide cellular re-organisation according to a physiological pattern.

**Fig. 4:**
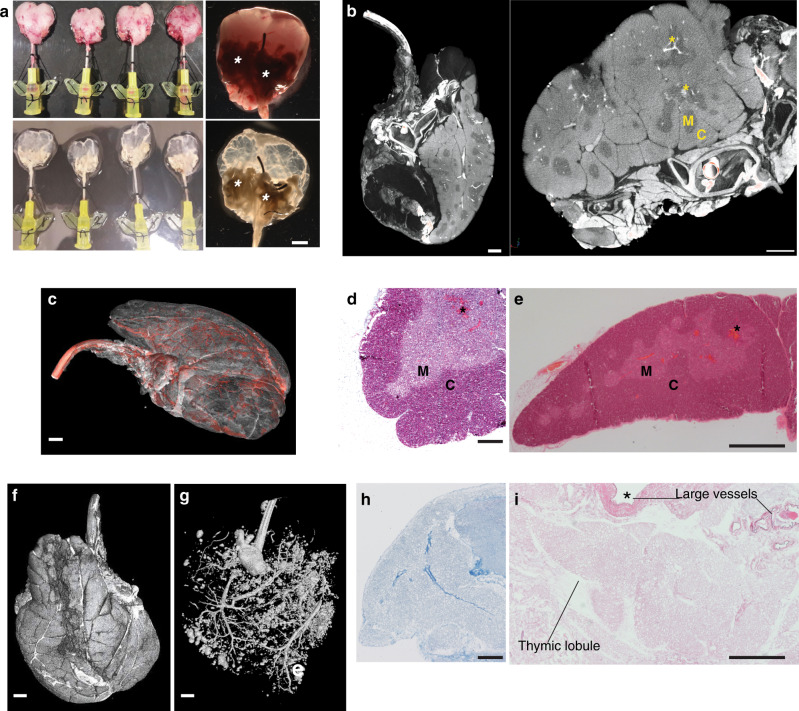
Whole-organ thymus perfusion and decellularisation. **a** Gross appearance of cannulated rat thymi before (upper panels) and after (lower panels) decellularisation. A 24G cannula is inserted into the carotid artery and used to perfuse the organ with detergent and enzymatic solutions. Asterisks indicate extra-thymic tissues that allowed connection between thymic tissue and cannula through the large blood vessels (*n* = 120). Scale bar, 2 mm. **b** Micro-CT images of cannulated rat thymus showing extra-thymic tissues, large blood vessels and the 24G cannula entering the artery. Iodine contrast shows clear demarcation between cortex (C, bright) and medullary (M) areas; blood vessels (asterisks) are represented by very bright areas between and inside the parenchyma (*n* = 3). Scale bar, 1.5 mm. **c** Micro-CT 3D image of whole rat thymus cannulated where iodine contrast shows vasculature tree (segmented in red, *n* = 2). Scale bar, 1.2 mm. **d** Masson’s trichrome stain of a fresh rat thymus lobe staining in red keratins, in blue collagen and in pink cytoplasm. C cortex, M medulla (*n* = 3 thymi). Scale bar, 250 μm. **e** Haematoxylin & Eosin (H&E) stain of a fresh rat thymus. C cortex, M medulla (*n* = 3 thymi). Scale bar, 500 μm. **f** Micro-CT image of cannulated rat thymus showing the whole 2 lobes in 3D. Scale bar, 1.2 mm. **g** Micro-CT image of a cannulated rat thymus injected with Microfil® and thresholded to demonstrate perfusion of both thymic lobes. Scale bar, 1.2 mm. **h** Masson’s trichrome stain of a paraffin section of a decellularised rat thymus scaffold demonstrating collagen fibres (blue) and absence of keratins, muscle fibres and cell cytoplasm (*n* = 3 scaffolds). Scale bar, 250 μm. **i** H&E of a decellularised thymus scaffold showing intact thymic lobule ECM and preservation of both large and small vasculature wall (*n* = 4 scaffolds). Scale bar, 500 μm.

The thymus develops from the third pharyngeal pouch^4^ as separate lobes which migrate into their final location as independent vascularised structures, after which the lobes adhere to each other through a layer of connective tissue. Owing to the lack of a main arterial supply to the whole-organ, it has not hitherto been possible to apply a perfusion-decellularisation approach to obtain ECM scaffold from the thymus, analogous to that achieved for many other organs^35^. Thus, we developed de novo an approach permitting whole-organ perfusion of the thymus. Briefly, one carotid artery was left open for subsequent cannulation to allow perfusion of the organ, while all other arteries (downstream of the emergence of vessels reaching the thymus) were closed, as shown by gross microscopy and as detailed in Methods section (Fig. 4a, upper panels).

We applied X-ray microfocus-computed tomography (Micro-CT) of cannulated whole rat thymi to allow 3D visualisation of the 24G cannula sited in the carotid artery (Fig. 4b). Micro-CT is a non-destructive technique that permits visualisation of the vasculature containing blood (white, dense areas) via iodine contrast; moreover, it facilitated clear demarcation of cortical (brighter areas, C) and medullary regions (M) through virtual segmentation, thereby demonstrating whole-organ 3D imaging of the thymus at high resolution (Fig. 4b; Supplementary Fig. 4a and Supplementary Movie 2). When the parenchyma was rendered more transparent, the 3D whole-organ micro-CT images allowed enhancement of the 3D thymic vascularisation segmented in red (Fig. 4c). Standard histology by masson trichrome (MT) and haematoxylin & eosin (H&E) stains confirmed the distribution of cortical and medullary areas and vascular (*) structures (Fig. 4d, e).

Importantly, we demonstrated that this microsurgical approach allowed perfusion of both thymic lobes by injecting a Microfil® compound through a single cannula, and, after solidification and fixation, we imaged the whole organ by Micro-CT (Fig. 4f, g). Perfusion through the cannula of MilliQ-H_2_O, followed by detergent and DNAse solutions allowed whole-organ decellularisation over a 6-day period (Fig. 4a, lower panels). MT and H&E stains demonstrated complete acellularity of the organ, which displayed a fine, reticular, collagen-enriched ECM and preservation of the macro- and micro-vasculature (Fig. 4h, i and Supplementary Fig. 4b, c). Thymic ECM was characterised by colorimetric staining for ECM proteins that demonstrated preservation of elastin while confirming the absence of intermediate filaments, including keratins (Supplementary Fig. 4d). Likewise, there was essentially no residual DNA after decellularisation (Supplementary Fig. 4e). Scanning electron microscopy (SEM) showed distribution of ECM fibres at high resolution (Supplementary Fig. 4f), and this was preserved in whole-organ scaffolds which were sterilised by γ-radiation and which could be stored in physiologic solution.

### Natural scaffolds facilitate functional thymic morphogenesis ex vivo

**Fig. 5:**
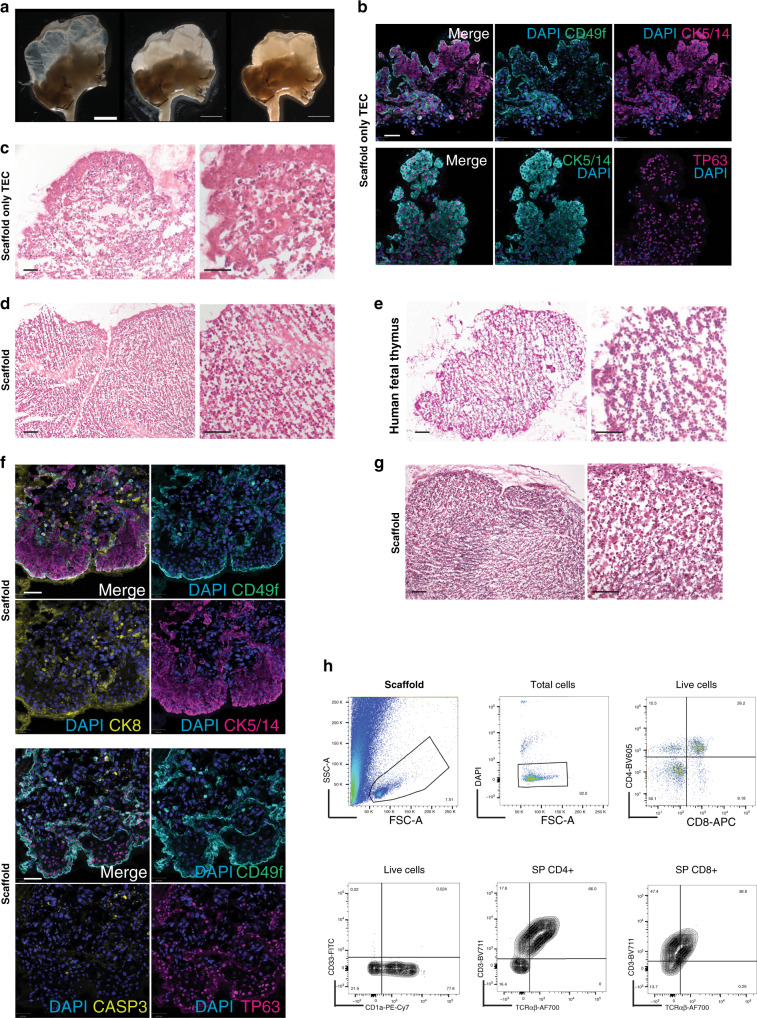
Functional repopulation of whole-organ thymus scaffolds. **a** Gross microscopy representative of a thymus scaffold before (left panel), soon after injection of stromal cells (middle panel) and following 4 days of culture (right panel). Thymic lobes from empty progressively increase density and get remodelled as shown by shrinking of the scaffold and increase tissue volume (*n* = 60 repopulated scaffolds). Scale bar, 4 mm. **b** Immunofluorescence labelling of thymic epithelial cells (TEC) grown within a decellularised scaffold demonstrating presence of CK5-14^+^, TP63^+^ and CD49f^+^ TEC. Nuclei are stained with DAPI (*n* = 4 repopulated scaffolds). Scale bar, 30 μm. **c** H&E staining shows histology of a scaffold repopulated only with TEC and cultivated for 5 days (*n* = 4 repopulated scaffolds). Scale bar, 50 μm. **d** Haematoxyin & Eosin (H&E) staining of a scaffold repopulated with both expanded clonogenic TEC and thymic interstitial cells (TIC) and cultivated for 5 days prior to fixation and histological analysis. Stromal cells reorganise along the scaffold with a pattern similar to the one (**e**) observed in early (9-week post-conception, wpc) human foetal thymus (*n* = 4 repopulated scaffolds and *n* = 2 human foetal thymi). Scale bar, 100 μm. **f** Immunofluorescence labelling of TEC seeded together with TIC and grown within a decellularised scaffold demonstrating CK5-14^+^ cells localised in the subcapsular region while CK8^+^ cells prevalently localised in the inner regions; TP63^+^ TEC were mainly CD49f^+^. Nuclei are stained with DAPI (*n* = 4 repopulated scaffolds). Scale bar, 100 μm. **g** H&E staining of a scaffold repopulated with TEC, TIC and haematopoietic progenitors after 7 days of culture (*n* = 4 repopulated scaffolds). Scale bar, 100 μm. **h** Representative FACS analysis (*n* = 6 repopulated scaffold in three independent experiments) of CD45-positive population isolated from repopulated scaffold seeded with triple negative (TN, CD3^−^CD4^−^CD8^−^) progenitors and co-cultured for 8 days. The FSC-A, SSC-A plot displays the presence of cells as well as of debris derived from the scaffold ECM during dissociation for release of haematopoietic cells (top left panel). Viable cells were ~90% of total cells (top mid panel). TN developed within the scaffold gave rise to double positive (DP) and single positive (SP) CD4 and CD8 expressing cells (top right panel, 5000 cells). Live cells were positive for CD1a and negative for CD33 (bottom right panel). CD4 and CD8 were positive for CD3 and express TCRαβ (bottom mid and left panel).

We next investigated the capacity of epithelial thymic cells extensively expanded as described above to repopulate and grow within a whole-organ 3D natural scaffold ex vivo. Scaffolds were injected with 4–6 million clonogenic TEC that had been expanded for 4–6 weeks. Repopulated scaffolds were then maintained in static conditions for up to 14 days in epithelial medium, over which time they showed scaffold remodelling and progressively increasing volumes (Fig. 5a). TEC re-organised along the 3D ECM structure from the subcapsular areas (CD49f^high^) into the innermost regions of the scaffold. Seeded TEC expressed CK5-14^+^ and/or CK8^+^; were in active cell cycle (MKI67^+^); were negative for the apoptotic marker, Caspase 3; and broadly expressed TP63 (Fig. 5b and Supplementary Fig. 5a). These data demonstrate that TEC were supported by the 3D ECM. Nevertheless, under these in vitro conditions and in the absence of any other cell type (Fig. 5c), seeded cells resembled TEC detected in patients affected by primary immunodeficiencies wherein the distribution of thymic stroma is severely perturbed^36^.

To address this issue, we next co-seeded expanded TEC together with cultured TIC. We optimised the TEC: TIC ratio at 5:1 in order to avoid interstitial cell overgrowth. Strikingly, after only 5 days of co-culture, the presence of TIC promoted a cordoned stromal organisation that largely phenocopied an early (9-week post-conception, wpc) foetal thymus (Fig. 5d, e). TIC were found to be crucial for re-organisation of TEC along the 3D ECM of the reconstituted scaffold. In the presence of TIC, cortical CK8^+^ TEC were mostly surrounded by a subcapsular-like layer of CK5-14^+^ TEC that expressed CD49f and TP63, phenocopying the native thymus (Fig. 5f and Supplementary Fig. 5b, c). SEM of repopulated scaffolds confirmed cell–cell and cell–matrix interactions between TEC, TIC and decellularised ECM, as well as the preservation of vascular structures (*) (Supplementary Fig. 5d).

We next considered it appropriate to compare the organised thymus stroma reconstituted in vitro from clonogenic cells with clinical thymus slices that are cultured on a sponge up to 21 days, in order to remove donor thymocytes, before transplantation into athymic patients^20,37^. The healthy status of the reconstituted stroma within our scaffolds seemed of particular relevance when considering that current clinical protocols for thymus slice preparation show disorganised stroma with variable survival after 14 and 21 days of culture, and equally importantly, residual CD3^+^ thymocytes that pose an overt risk of graft-versus-host disease for a transplant recipient (Supplementary Fig. 5e).

In order to assess the functional potential of the 3D thymic microenvironment, natural scaffolds were repopulated with TEC and TIC as described, and cultivated for 4–6 days before being injected with thymocyte precursors. Given the size (0.5–1 cm^3^) of the repopulated scaffolds, we cultured cells for a relatively short-term (up to 2 weeks) insufficient time vis-a-vis 5–6 weeks required for haematopoietic stem cell (HSC) lineage differentiation^38^. To accommodate this, scaffolds were repopulated with flow cytometry-sorted triple negative thymocytes (TN, CD3^−^CD4^−^CD8^−^) characterised for the expression of early lymphoid markers CD1a, CD5, CD7 and CD31 (Supplementary Fig. 5f, g). Immune cells within repopulated scaffolds were negative for CD33, positive for CD1a and could differentiate toward both double (DP) and mature single positive (SP) CD4^+^ and CD8^+^ stages, resulting in a ratio of CD4^+^:CD8^+^ cells that phenocopies the ratio found in the native human thymus (Fig. 5h). EpCAM^+^ TEC within the scaffolds expressed also HLA-DR which was not detected on TEC expanding in culture (Supplementary Fig. 6a–c). In contrast, TN injected into scaffolds containing TIC-only stroma did not survive (Supplementary Fig. 6d). In conclusion, it was possible to cultivate organ-size, 3D structures that supported the survival and appropriate spatial organisation of thymic stromal cells, that in turn facilitated thymocyte differentiation.

### Human thymus morphogenesis recapitulated in vivo

We next investigated whether repopulated thymus scaffolds might mature and sustain human T cell reconstitution from HSC in vivo. To this end, scaffolds that had been repopulated and cultured in vitro for 5–6 days, were implanted subcutaneously into humanised NOD.*scid*.Il2Rγc^*null*^ (NSG) mice. NSG mice were sub-lethally irradiated and transplanted with highly purified CD34^+^ HSC from either human cord blood (CB CD34^+^) or foetal liver (FL CD34^+^) 4–6 weeks before they were implanted with thymus scaffolds co-seeded with stromal cells (TEC and TIC) and CD34^+^ HSC at defined ratios (Supplementary Fig. 7a). Note that the HSC, which can act as sources of human myeloid cells, were added to the grafts to accelerate thymus development, but were not essential (see below).

**Fig. 6:**
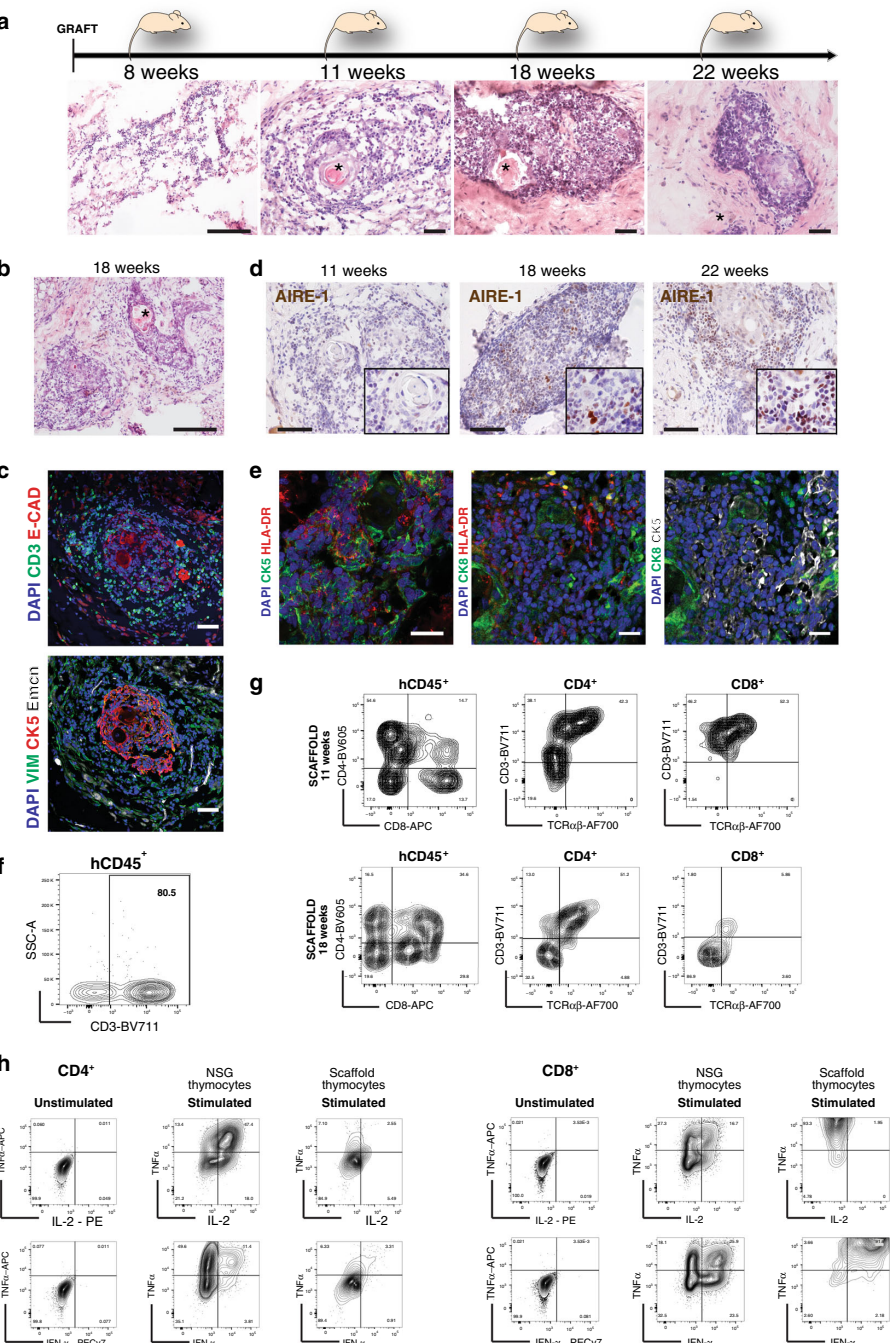
Repopulated thymus scaffolds mature in vivo and promote functional T cell development. **a** H&E staining of histological sections of thymic scaffolds harvested at different time points post-transplant (8, 11, 18 and 22 wpt). Asterisks indicates Hassall’s Bodies (HB); n = 18 scaffolds in 3 independent experiments. Scale bar, 100 μm. **b** H&E of histological section of a thymic scaffold not seeded with CD34^+^ HSC at grafting and harvested at 18 wpt; Asterisks indicates Hassall’s Bodies (HB). Scale bar, 100 μm. **c** Immunofluorescence analysis for CD3, E-Cadherin, CK5, Vimentin (VIM) and mouse Endomucin (Emcn) at 11 wpt for thymic scaffolds repopulated with stroma and CD34^+^ HSC; *n* = 4 scaffolds in two independent experiments. Scale bar, 50 μm. **d** Immunohistochemistry for AIRE-1 of thymus scaffolds harvested at 11, 18 and 22 wpt demonstrate progressive increase of AIRE-1^+^ cells; *n* = 8 scaffolds in three independent experiments. Scale bar, 50 μm. **e** HLA-DR was detected in cytokeratin-positive medullary (CK5) and cortical (CK8) cells in repopulated scaffolds by immunofluorescence; *n* = 18 scaffolds in three independent experiments. Scale bar, 25 μm. **f** Representative FACS analysis of a dissociated thymic scaffold at 11 wpt showing presence of CD3^+^ cells (80% of total human (h) CD45^+^ population; *n* = 9 scaffolds). **g** FACS analysis of dissociated thymic scaffolds demonstrates both double positive (DP) and single positive (SP) CD4 and CD8 cells. SP CD4 and CD8 cells also expressed CD3 and TCRαβ, demonstrating the presence of immature single positive (ISP) as well as fully mature thymocytes at both 11 wpt and 18 wpt time points (*n* = 9 scaffolds, live cells = 1100–3800). **h** Representative FACS analysis of sorted and in vitro expanded CD3^+^ cells. CD3^+^ cells were sorted either from repopulated thymus scaffolds or from the endogenous NSG thymus 18 wpt, expanded in vitro prior to FACS analysis that shows presence of CD4^+^ and CD8^+^ SP cells. Expanded CD4^+^ and CD8^+^ cells were stimulated by PMA-ionomycin and intracellular cytokine staining performed: CD4^+^ and CD8^+^ isolated from endogenous mouse thymus were able to produce IL2, TNFα and IFNγ, while SP CD4 and CD8 cells developed within thymus scaffolds were able to produce only limited amount of IL2. CD8 SP cells, though in minor number in each scaffold compared to CD4^+^ were able to produce the highest level of IFNγ and TNFα; *n* = 2 independent experiments (live cells from scaffolds = 7000 and from endogenous thymus = 52000).

Decellularised natural scaffolds previously seeded and cultivated in vitro, were grafted subcutaneously into NSG mice. At least two scaffolds were grafted into each mouse. Grafts were individually harvested at 1, 2, 8, 11, 18 and 22 weeks post-transplantation (wpt; Fig. 6a and Supplementary Fig. 7a, b). Early time points were important to confirm stromal cell survival and re-organisation. At 8 wpt, stroma (TEC and TIC) seeded in natural scaffolds still showed a cordon-like distribution or compact epithelial structures by contrast to the unique 3D thymic epithelial reticulum (Fig. 6a). However, by 11 wpt, ordered morphogenesis had progressed further, and more mature thymic structures were consistently detected among scaffolds seeded with stroma and CD34^+^ HSC. Among those were structures very similar to Hassall’s Bodies (HB), an anatomic trait of human thymus that is much less evident in mouse (Fig. 6a, b HB indicated by asterisk and Supplementary Fig. 7c).

Immunohistochemistry (IHC) for the epithelial marker E-Cadherin (ECAD, red) and for the T cell receptor (TCR) (anti-CD3; green) demonstrated thymic morphogenesis with epithelial cells forming HB and interacting with CD3^+^ cells from the scaffold (Fig. 6c). Epithelial (CK5^+^) and mesenchymal (Vimentin^+^CK5^−^) cells, and angiogenesis (as detected by Endomucin immune-staining) were detected in scaffold transplants (Fig. 6c, lower panel; Supplementary Fig. 7d). In some experiments VeraVec^39^ endothelial cells were seeded in addition to cultivated TEC and TIC since they were reported to favour faster revascularisation in reconstituted bone marrow (BM) niches^40^. However, thymus morphogenesis and T cell development occurred independently of the presence of human endothelial cells at seeding, probably owing to murine vascularisation of the scaffold.

To ask whether the reconstituted scaffolds attracted circulating haematopoietic progenitors, we examined morphogenesis at 18–22 weeks post-transplantation (wpt), and found that thymic maturation was occurring in most repopulated scaffolds (Fig. 6a) including those that were not injected with HSC prior to grafting (Fig. 6b). Moreover, AIRE expression progressively increased from 11 wpt to 22 wpt (Fig. 6d), and within several areas, with or without HB, there were scattered CD11c^+^ dendritic cells (DC), with HLA-DR expression detected by IHC on DC and by IHC and FACS on TEC (Fig. 6e and Supplementary Fig. 7e, f).

In order to functionally characterise the implanted scaffolds, grafts were harvested, dissociated into single cells and analysed by flow cytometry. CD3^+^ cells were the prevalent phenotype within the hCD45^+^ population within the re-engineered thymic implants. The high percentage (30–80%) of hCD45^+^ cells within each scaffold that were CD3^+^ indicates that the haematopoietic progenitors were subjected to an instructive thymic microenvironment provided by cultivated human stromal cells (Fig. 6f), since BM of the same mice were by contrast primarily repopulated with CD33^+^ myeloid and CD19^+^ B lineage cells (Supplementary Fig. 8a).

Flow cytometry showed that scaffolds were able to support thymocyte development (77.5% of all implanted scaffolds), with SP CD4^+^ and CD8^+^ cells expressing TCRαβ (Fig. 6g;). Of note, the CD4:CD8 ratio for cells maturing in scaffolds favoured CD4^+^ SP cells (~3:1), a feature of human thymocyte development that is not seen when circulating human BM-derived progenitors undergo differentiation within the endogenous thymi of NSG mice (Supplementary Fig. 8b). Immature SP (ISP) and DP thymocytes were also present in different proportions within the scaffolds, demonstrating ongoing thymopoiesis, with consistent representation of DP cells (Fig. 6g and Supplementary Fig. 8c). Nonetheless, their reduced presence by comparison to those reported in human thymi examined directly ex vivo may reasonably reflect the fact that further optimisation will be required to establish the precise schedules and kinetics of differentiation in the native organ. In contrast, no CD3^+^ cells were retrieved from scaffolds repopulated with TIC, VeraVec, HSC CD34^+^ (without TEC) at 11 wpt or empty scaffolds at 22 wpt (Supplementary Fig. 8d, e).

We sorted hCD45^+^CD3^+^ cells from thymus scaffolds and from the endogenous thymi of NSG mice at 18 wpt, and then interrogated the cells’ functional potential in vitro. CD3/CD28 Dynabeads® were used to induce the proliferation of sorted cells for from 12 to 28 days until sufficient cells were obtained for functional studies. Interestingly, the respective CD4/CD8 ratios of scaffold-associated or endogenous thymus-associated cells were sustained upon expansion in culture, indicating that this property had been differentially “instructed” by human and mouse thymic stroma. When stimulated in vitro with phorbol myristate acetate (PMA) and ionomycin (Io), the expanded CD4^+^ and CD8^+^ cells could produce IFNγ and TNFα, whereas IL2 was produced to a much lesser extent from cells developing in the human scaffolds than by T cells derived from the NSG thymi, (Fig. 6h). Of note, thymocytes freshly isolated from human thymus also respond to PMA-Ionomycin with low IL2 production (Supplementary Fig. 8f)

Finally, we interrogated the capacity of repopulated scaffolds to support peripheral T cell reconstitution in an athymic NSG-*Foxn1*^*null*^ (NSG-nude) mouse where the scaffolds were the only thymic stroma. NSG-nude mice were humanised 4–8 weeks prior to grafting. Littermate NSG (Hairy) mice were utilised as positive control for BM and peripheral repopulation (spleen; Supplementary Fig. 9a, left panels). Upon BM reconstitution no T cells (hCD3^+^) were detected in the periphery of these mice when transplanted with empty scaffolds (Supplementary Fig. 9a, middle panels). On the contrary, when NSG-nude mice were grafted with scaffolds previously repopulated in vitro with human thymic stroma, hCD3^+^ cells were detected in the periphery (Supplementary Fig. 9a, right panels). Of note, hCD45^+^CD3^+^ cells from the spleens of NSG-nude mice were purified at two time points (10 wpt and 18 wpt) and analysed for expression of specific genes supporting TCR-activation including *TRBC1/2, TRAV19, NT5E, CD45RA, TRAC, CD69, PTPN6 (SHP-1), PTPRC, PPIA, CD22* and *LTB*^41–49^ (Supplementary Fig. 9b).

## Discussion

The data described in this study demonstrate that postnatal human thymus harbours epithelial (TEC) and interstitial cells (TIC) that can expand to clinically relevant numbers in vitro, suitable for contributing to the reconstruction of a human functional thymus in vivo. This is an important proof-of-principle that a long-lived phenocopy of a human thymus utilising only postnatal, cultivated cells is achievable.

Our cell sorting strategy for isolating medullary and cortical subpopulations showed that both mTEC and cTEC expressing CD49f are enriched for clonogenic TEC with the ability to be subcultured and to be significantly expanded ex vivo. Interestingly, the expanding mTEC and cTEC mostly lost the signatures of their respective compartments of origin, seemingly consistent with there being a common progenitor present throughout thymic tissue^5,16,50^. Unexpectedly, clonogenic TEC co-expressed genes and also displayed some behaviour typical of mesenchymal cells: to our knowledge, this hybrid phenotype is an unique, cell-intrinsic feature of thymus stroma that is stably maintained over many passages in culture. A systematic study based on single-cell clonal analysis, which represents the paradigm of epithelial stem cells in vitro^51,52^, will offer the means to further dissect the complexity and the properties of these thymic clonogenic cells.

Our combined approach of single-cell transcriptome analysis with the prospective isolation of medullary and cortical subpopulations was crucial to identify clusters of cells common to both medulla and cortex (comTEC) among the highly heterogeneous cell types present in the two compartments. The transcriptional profile of the comTEC defined a common medullary and cortical signature in vivo, including an EMT profile, that were overtly recapitulated by the clonogenic TEC in vitro. This feature of the thymus epithelium may be explained by the fact that a 3D reticular type of epithelial stratification occurs solely in the thymus, but future work should certainly address the molecular mechanisms that guide this cell-intrinsic property.

Additionally, a distinct, bona fide interstitial cell population (TIC) could be extensively expanded in vitro and shared several features with other mesenchymal stromal cells and pericytes. Those cells were important in guiding TEC during morphogenesis, both ex vivo and upon transplantation in vivo since morphogenesis was defective in their absence. Possibly their role extends beyond supporting TEC development and regeneration^53^, to synergising with TEC in directly regulating T cell development^54,55^. Further development of this organ reconstruction system will offer the opportunity to address systematically the reciprocal and synergistic roles of TEC, TIC, endothelial and other human stromal cells (e.g. dendritic cells) during both morphogenesis and thymopoiesis.

Of note, 3D natural ECM were shown to be crucial for thymus morphogenesis from cultivated cells. To date, it has only been possible to obtain thymus scaffolds by mechanically shaking or rotating the organ in a detergent solution^13^. More recently, it was demonstrated that native thymic ECM improved re-aggregation thymic organ cultures in vitro and their T cell output in vivo^15^. Both these approaches were based on applying to the mouse thymus a combination of ionic and non-ionic detergents which may not be practically applicable to human thymus required for clinical translation. Instead, the whole-organ perfusion-decellularisation approach that we describe allows preservation of the fine 3D ECM meshwork that is crucial for both ex vivo morphogenesis and long-term reconstitution in vivo. Moreover, human cultivated TEC gave rise to Hassall’s Bodies, thus demonstrating their capacity to maintain key species-specific characteristics not previously reported for thymic progenitor differentiation in vivo^56,57^.

Functional gene products such as AIRE and HLA-DR are also downregulated in the thymus stroma of human SCID patients in vivo^58^, indicating that their expression is dependent on a complex microenvironment which includes crosstalk between the stroma and thymocytes. Hence, the conspicuous re-expression of AIRE and HLA-DR that we observed attests to the degree of reconstitution achieved in our system.

Crucial to demonstrating the functional competence of TEC and TIC upon in vitro expansion was their capacity to attract circulating human HSC, support T cell development and repopulate the periphery of athymic, NSG-nude mice. Species-specific stroma in an organ such as the thymus is crucial to its own function; indeed, we consistently achieved a physiologic human CD4^+^/CD8^+^ ratio that was not observed with hybrid mouse-human in vitro systems of human T cell development^14,38^. Importantly, human T cells that developed in vivo within the human scaffolds were functionally different in their response to stimulation from those which developed in the mouse thymus, demonstrating the instructive capacities of the human stroma.

In conclusion, we have used a multidisciplinary approach to generate an in vivo, long-lasting thymus where both the haematopoietic and stroma compartments are of human origin. Such a system opens the possibility of addressing very many immunological questions including the development and functional maturation of conventional and unconventional human T cells (e.g. Treg, NK, γδ); positive and negative selection of MHC-I and II-restricted human T cells; and the roles of additional factors in the establishment and maintenance of tolerance. Future work and further optimisation will be needed to estimate the full potential and limitations of our system in relation to each of these crucial questions. Notwithstanding, the successful completion of these steps can allow medically relevant applications such as thymus transplantation in primary immune deficiencies as athymic DiGeorge syndrome and *Foxn1*^*null*^ (nude) babies; the control of tolerance in congenital conditions as autoimmune polyendocrinopathy-candidiasis-ectodermal dystrophy (APECED) patients, and in immunosuppression-free organ transplantations.

## Methods

### Animals

All animal procedures were in accordance with ethical approval and UK Home Office Project License (PPL) 70/8904 and PDD3A088A. NOD.Cg-*Prkdc*^*scid*^.I*l2Rγc*^*tm1Wjl*^ (NSG) and NOD.Cg-*Foxn1*^*em1Dvs*^*.Prkdc*^*scid*^*.Il2R*γc^*tm1Wjl*^ (NSG-Nude, Stock No: 026263) were obtained from Jackson Laboratory, re-derived and maintained at The Francis Crick Institute’s biological resource facility or at the UCL biological unit. Mice were maintained on a 12-h light–dark cycle, ambient temperature 19/22 °C, and humidity 45/65%. Sprague Dawley rats were kept in the UCL breeding facility.

### Human tissues

Postnatal thymi were donated by patients (age range 3 days to 11 years old) undergoing cardiothoracic surgery at the Great Ormond Street Hospital. A written informed consent was obtained from the patient parents or legally authorised representatives under ethical approval (REC No 15/YH/0334 and 07/Q0508/43-06-MI-13 (B)). Human foetal livers (FL) were provided by the Joint MRC/Wellcome Trust Human Developmental Biology Resource (HDBR) under informed ethical consent with Research Tissue Bank ethical approval (REC No 08/H0712/34+5 and 08/H0906/21+5). Umbilical Cord Blood (CB) was obtained from normal full-term deliveries after signed informed consent. CB sample collection was approved by the East London Ethical Committee (REC No 06/Q0604/110) and in accordance with the Declaration of Helsinki.

### Clinical thymus slices preparation and culture

After capsule removal, 8–15 g of post-natal thymus tissue was processed with Stadie-Riggs microtome (Thomas Scientific), in order to obtain 1 mm thick slices. Slices were individually mounted on nitrocellulose filters (Millipore), subsequently placed on Spongostan surgical sponges (Ferrosan Medical Devices) and bathed in culture medium (F12 (Gibco), 10% Heat-Inactivated Fetal Bovine Serum (Gibco) and 1% Penicillin/Streptomycin (Sigma)) in 10-cm Petri dishes up to 21 days; culture media was replaced every day.

### CD34^+^ HSC purification and sorting

Mononuclear cells (MNCs) from two up to five CB collections were pooled and purified by Ficoll-Paque density centrifugation (GE Healthcare Life Sciences, Buckinghamshire, UK) followed by ammonium chloride red cell lysis. Density-separated CB MNCs were magnetically sorted for CD34 positivity via the EasySep Human CD34 Positive Selection kit (Stemcell Technologies) according to the manufacturer’s instructions; alternatively, they were sorted by fluorescent activating cell sorting (FACS). Foetal liver (FL) CD34^+^ were isolated from human foetal tissue ranging from 12 to 20 weeks post-conception (wpc). Tissue digestion was performed at 37 °C using an enzymatic solution (0.1 U/mL Collagenase A (Roche), 0.8 U/mL Dispase II (Roche) and 100 μg/ml DNase I (Roche) in RPMI, 2% FBS and 1% Penicillin/Streptomycin). Cells were pelleted and processed for ammonium chloride red cell lysis. FL MNCs were magnetically sorted for CD34 positivity.

### Thymocyte isolation and sorting

Human thymocytes were obtained by mechanical tissue dissociation of postnatal thymi. Enrichment of triple negative (TN, CD3^−^CD4^−^CD8^−^) cells was achieved by magnetic sorting using biotinylated anti-CD3, anti-CD4, anti-CD8a and anti-CD235ab antibodies (BioLegend) and Magnisort SAV negative beads (Invitrogen). The negative-selected fraction was processed for FACS purity sorting (CD11c^−^CD19^−^CD56^−^CD3^−^CD4^−^CD8^−^ cells; FACSAria III machine, BD Bioscience; FACSDiva 8.0.1 software).

### Isolation and cultivation of thymic stromal cell

We assessed human thymi obtained from 33 patients ranging in age from 3 days to 11 years who underwent open-chest cardiac surgery. All thymi, independent of donor age, contained clonogenic TECs generating colonies that could be expanded extensively upon weekly passage. Thymic tissue fragments were dissociated to single cells by enzymatic treatment (0.4 mg/mL Collagenase D (Roche), 0.6 mg/mL Dispase II (Roche), 40 μg/mL DNAse I (Roche)) for ~30–45 min at 37 °C. Cells were pelleted and re-suspended for cell counting. Freshly dissociated thymic cells were plated over a layer of lethally irradiated mouse fibroblast (3T3-J2). Alternatively, thymus single-cell suspension was depleted of CD45 and CD235ab (red blood) expressing cells by immunomagnetic separation. The enriched fraction was stained and sorted using FACSAria III machine (FACSDiva 8.0.1 software) for cortical (EpCAM^low^CD205^+^) and medullary (EpCAM^high^CD205^−^) thymic epithelial cells (TEC) prior to cell culture over 3T3-J2 feeders.

Epithelial cells expanded in cFAD medium composed by a mixture of 3:1 of DMEM-1X (Gibco) and F-12 Nut Mix (Gibco), supplemented with 10% Fetal Bovine Serum (Sigma), 1% penicillin and streptomycin (100X, Sigma), Hydrocortisone (0.4 µg/ml, Calbiochem), Cholera Toxin (10^−10^ M, Sigma), Triodothyronine (T3) (2 × 10^−9^ M Sigma) and Insulin (5 µg/ml, Sigma). Human epithelial growth factor (hEGF, 10 ng/ml, PeproTech) was added after 3 days of culture and then every other day at each feeding. TEC were plated with a density of 2000–4000 cells/cm^2^, incubated at 37 °C and 6% CO_2_ reaching confluence between 5 and 7 days of culture^19,50^. TEC colony-forming efficiency (CFE) assay was performed every other passage. Five-hundreds cells were obtained by a serial dilution and plated over lethally irradiated 3T3-J2 feeders in 60 mm tissue culture dishes. After 12 days of culture, growing colonies were fixed in 4% Paraformaldehyde (PFA) and stained with Rhodamine-B (1%, SIGMA-ALDRICH) for 15 min. Colonies were scored under a dissecting microscope.

Interstitial cells of the thymus (TIC) were derived either by sorting EpCAM^−^ stroma-enriched thymic population or by explants of human thymus. Fragments of thymic tissue were placed on 60 mm dishes pre-coated with Matrigel^TM^ (Corning), diluted 1:100 in Megacell medium (Sigma). Fragment were left to adhere for 30 min and then gently covered with Megacell medium supplemented with 2.5% FBS HI (Life Technologies), 1% Penicillin/Streptomycin (Sigma), 1% l-glutamine (Life Technologies), 1% Non-Essential Aminoacids (Life Technologies), 100 mM beta-Mercaptoethanol (Life Technologies) and basic-FGF (Sigma). After 7 day of culture, the cells outgrown from the explants were harvested, cultured at 37 °C in a 6% CO2 and 5% O2 atmosphere and passaged every 3–4 days, when TIC reached confluence^33^.

### Lymphocyte culture and in vitro stimulation assay

Human CD45^+^/CD3^+^ sorted cells were cultured in RPMI 1640 (Gibco), Glutamax (Life Technologies) with 10% Foetal Bovine Serum (FBS, Life Technologies), 1% Penicillin/Streptomycin (Life Technologies) and supplemented with human recombinant IL2 (50 U/mL, R&D system) and IL-7 (5 ng/mL, Invitrogen) up to 28 days in 96, 48 and 24 wells (Falcon). For expansion, anti-CD3/CD28 beads (Thermofisher) were added in the proportion of 1:1.

Cytokine production was assessed by Phorbol Myristate Acetate (PMA) and Ionomycin (Io) stimulation assay: human CD45^+^/CD3^+^ sorted T cells and freshly isolated human thymocytes were incubated in RPMI, supplemented with 20% Human Serum Heat Inactivated (SIGMA-ALDRICH), Protein Transport Inhibitor Cocktail (500X, eBioscience), PMA (40 ng/mL, SIGMA-ALDRICH) and Io (4 μg/mL, SIGMA-ALDRICH) for 6 h at 37 °C. T cells were then washed with HBSS solution and stained for CD3, CD4 and CD8 (BioLegend), as well as APC-Cy7 fixable viability dye (Invitrogen), before fixation and permeabilisation with intracellular staining buffer kit (BioLegend) and intracellular staining with antibodies anti- IFNγ, anti-TNFα and anti-IL2 (BioLegend). Expanded and unstimulated T cells were used to set the FACS gates.

### Endothelial cells (HuVEC-VeraVec) culture

Human endothelial cells (Angiocrine, CAT: HVERA101) were cultivated on a layer of 0.1% gelatine (Merck) in Medium 199 (Gibco) supplemented with 20% Foetal Bovine Serum, FBS (Gibco), 1% Antibiotic/Antimicotic (ThermoFisher), 10 mM HEPES (Gibco), 100 μg/ml Heparin (SIGMA-ALDRICH) and 50 μg/ml endothelial supplement ECGS (Millipore).

### Flow cytometry analysis

Single-cell suspensions were stained with ad hoc antibody mix (Supplementary Table 1) in Hanks Balanced Salt Solution (HBSS, Life Technologies) supplemented with 2% FBS (Life Technologies) for 30 min on ice. DAPI (SIGMA-ALDRICH) or Zombie Live-Dead dye (Invitrogen) was used to discriminate live from dead cells. FACS phenotypic analysis was performed using Fortessa X-20 machine (BD FACSDiva8.0.1 software) and FlowJo^TM^ software (BD Bioscience). Gating strategies are detailed in the Supplementary Fig. 10.

### RNA isolation and RT-qPCR

Cultured and freshly isolated cells were collected for gene expression analysis in either BL-Buffer from ReliaPrep™ kit (Promega) or in Trizol TRI Reagent (SIGMA-ALDRICH) following the manufactures instructions. Precipitated and dried RNA was re-suspended in nuclease-free water (Qiagen). RNA concentration was measured using Nanodrop1000 (ThermoScientific) and RNA integrity (RIN) was evaluated using BioAnalyzer 2100 (Agilent). If needed, RNA was amplified using Ovation® RNA Amplification System V2 (Nugen) following the manufacturer’s instructions. Alternatively, RNA was converted into cDNA with GoScript™ Reverse Transcriptase kit (Promega) according to the manufacturer’s protocol. cDNA concentration was adjusted to 10 ng/μl. Quantitative (q)PCR was performed using PCR master mix (PrecisionPLUS-R - Primerdesign Ltd) with low-ROX and Taqman qPCR probes (Integrated DNA Technology, Supplementary Table 2) in MicroAmp Fast Optical 96 well Reaction Plates (Applied Biosystems) using the QuantStudio 3 Real-Time PCR System (Applied Biosystems).

### Single-cell RNA sequencing – 10X genomics

The cell numbers were confirmed using an Eve automated cell counter (NanoEnTek). Where possible an appropriate volume for 10,000 cells was made up to 46.6 μl with nuclease-free water. For lower cell concentrations 46.6 μl of cell suspension was loaded without further dilution. Reverse transcription and library construction were carried out following the Chromium single-cell 3′ reagent v3 protocol (10x Genomics) according to the manufacturer’s recommendations. Total complementary-DNA synthesis was performed using 12 amplification cycles, with final cDNA yields ranging from ~3 ng/μl to 15 ng/μl. The 10x Genomics sequencing libraries were constructed as described and sequenced on an Illumina HiSeq 4000, with read lengths of 28-8-98.

### Bioinformatics data analysis of single-cell data

Raw sequencing data were processed using the CellRanger pipeline (10x Genomics). Count tables were loaded into R and further processed using the Seurat 3R package^59^. We removed all cells with fewer than 200 distinct genes observed or cells with more than 20% (samples mTEC1, mTEC2) or 30% (samples cTEC1, cTEC2) of unique molecular identifiers stemming from mitochondrial genes. Principal component analysis was then performed on significantly variable genes and the first 20 principal components were selected as input for clustering and UMAP based on manual inspection of a principal component variance plot (‘PC elbow plot’). Clustering was performed using the default method from the Seurat package, with the resolution parameter set to 0.5. Area under the curve (AUC) summary intensity plots were prepared using the R-package AUCell.

### Bulk RNA sequencing library preparation and analysis

Total RNA was extracted from cultured cells using the RNeasy Micro Kit (Qiagen) and RNA total concentration was measured with a Nanodrop spectrophotometer. Total RNA was depleted of rRNA with Ribo-Zero, retrotranscribed using Superscript II reverse transcriptase (Invitrogen) and purified with RNA Clean Beads e e Ampure XP beads (Beckman Coulter).

Libraries were prepared with the TruSeq Stranded Total RNA Sample Preparation Kit (Illumina) starting from 100 ng of RNA per sample and sequenced with the Illumina NovaSeq 6000 machine, paired ends, and a coverage of 35 million reads.

RNA-Seq data were aligned with Salmon (GRCh38) and processed as follows. DEGs lists generation: analyses were performed with edgeR (v. 3.24.3). Significant DEGs were selected applying a 0.05 cutoff on FDR and a threshold of 1.5 on logFC. Genes comparison between different cell types: counts were normalised with the edgeR’s function cpm() with the argument log=T. Then, data were visualised in a heatmap generated by the function heatmap.2() (gplots v.3.0.1.2).

### NanoString analysis

hCD45^+^CD3^+^ cells were sorted from the spleen of two NSG-nude mice at 10 and 18 wpt (8081 and 1869 sorted events, respectively), lysed and hybridised to a chimeric antigen receptor-T cell panel for profiling 780 human genes at 65 °C overnight (NanoString Technologies). Hybridised samples were processed on an nCounter® prep station and data were collected on an nCounter® digital analyser (NanoString) following manufacturer’s instructions. Raw data were imported into nSolver4.0 (NanoString) for data quality checks, background thresholding and normalisation. The 6 spiked-in RNA Positive Control and the 8 Negative controls present in the panel were used to confirm the quality of the run. Background level was determined by mean counts and 2× s.d. of negative control probes. Samples that contained fewer than 50% of probes above background or that had imaging or positive control linearity flags were excluded from further analysis. Probes that have raw counts below background in all samples were excluded from differential expression analysis to avoid false-positive results. Data were normalised by geometric mean of 10 housekeeping genes present in the CAR-T Characterisation panel.

### Histology

Human thymic tissues and scaffolds samples were fixed (for 2 h to overnight) in 4% PFA and processed for either cryo- or paraffin-embedding. For cryo-embedding, fixed tissue was equilibrated in sucrose 25% and embedded in O.C.T. compound (VWR). Cryosections (thickness, 7 µm) were cut on a Leica Cryostat 3050. For paraffin-embedding, a Leica PelorisII tissue processor and Sakura Tissue-Tech embedding station were used. Paraffin section (thickness, 3–5 µm) were produced using ThermoFisher rotary microtome. Cryo- or Paraffin sections were stained with haematoxylin-eosin using an automatic station (Tissue-Tek Prisma), Masson’s Trichrome Kit (Leica, Raymond A Lamb, BDH Chemicals) and Van Gieson Staining Kit (Millipore-Merck).

### Immunostaining

Tissue sections or coverslips were fixed in 4% PFA, blocked and permeabilised simultaneously using a solution of 5% Normal Donkey Serum (NDS, Jackson Immuno Research) in PBS, containing 0.5% of TritonX (TritonTMX-100, SIGMA-ALDRICH). Tissue sections or cells were incubated with primary antibodies 5% NDS, 0.01% TritonTMX solution overnight at 4 °C. Secondary antibodies were incubated at room temperature for 45 min. Nuclei were counterstained with Hoechst 33432 (10−6 M) or DAPI present in the Fluoroshield^TM^ Mounting Medium (Abcam). List of primary and secondary antibodies used are listed in the Supplementary Table 3.

### Imaging

Phase contrast images of cultivated cells were acquired using an Olympus CK40 inverted microscope and Olympus SC50 camera. Live-imaging of cultured cells was acquired by scope Zeiss Axiovert 135 with Hamamatsu Orca R2 camera (objectives ×10 NA 0.25 Plan Neofluar).

For histological images Zeiss Axioplan2 microscope with Zeiss Axiocam HRc colour camera was used. Gross microscopy of organs was imaged using Zeiss StREO Discovery.V20 microscope and Zeiss Axiocam506 colour camera. Zeiss LSM710 inverted confocal microscope and Zen Black software was used to acquire immunofluorescence images. Confocal images were processed using Fiji and Improvision Volocity LE software.

### Vascular microsurgery, perfusion and decellularisation

Male rats ranging from 150 to 220 g in weight were used as source of thymus organs. The thymus lacks a common artery to supply blood to the whole organ, therefore it is not possible to perfuse it by inserting a single cannula in the main blood vessel as for other organs^35^. We developed a microsurgical approach to obtain whole-organ thymus perfusion which has been patented (Patent filing number UK1807788.3). Briefly, the vessels supplying blood to each lobe, were closed with a silk suture thread (size 6.0, F.S.T.) to seal the thymus in the following order: the right common carotid artery, the right subclavian artery, the right internal mammary artery, the right costocervical trunk, the right aortic arch, the left aortic arch, the left costocervical trunk and the left internal mammary artery. The left carotid artery was left open for subsequent cannulation and perfusion of the organ. Cannulated thymi were decellularised by perfusion of detergent-enzymatic treatment (DET). Organs were perfused with dH2O (18.2 mΩ/cm) for 96 h at 4 °C (0.2 ml/min) using a i150n peristaltic pump (*i Pumps*). DET was performed with 4% sodium deoxycholate (SDC; SIGMA-ALDRICH) at room temperature and 0.1 mg/mL DNase-I (SIGMA-ALDRICH) in 2.5 mM MgCl_2_ (SIGMA-ALDRICH). Decellularised rat thymi (scaffolds) were gamma-irradiated with a dose of 1780 Gy and stored in PBS at 4 °C for several weeks.

### Microfocus compute tomography

Rat thymi were extracted, cannulated and fixed in paraformaldehyde prior to immersion in potassium triiodide (I_2_KI) contrast in a 1:1 ratio with the formalin solution, which was injected into the cannula, prior to immersion of the thymus into a falcon tube containing the same solution (~20 ml volume). I_2_KI with a total iodine content of 63.25 mg/mL (iodine mass of 2.49 × 10^−4^ mol/mL) was used. Specimens were immersed in the contrast solution for 48 h prior to scanning. Immediately prior to micro-CT examination, the specimens were rinsed in distilled water to remove excess surface iodine and padded dry using gauze. They were wrapped in Parafilm®M and secured in a low-density plastic cylinder. Isotropic voxel sizes varied according to the geometric magnification achieved (inversely correlated with specimen size) and ranged between 5 and 9 µm. X-ray images were acquired using an XT H 320 microfocus-CT scanner with a multi-metal target (Nikon Metrology, Tring, UK). A tungsten target was used with an accelerating voltage of 100 kV and current of 100 microamps. Following micro-CT examination (duration of ~90 min), the specimens were fixed in 10% formalin to prevent tissue degradation and aid with the removal of iodine prior to macroscopic examination. Images were reconstructed using CT Pro 3D (Nikon Metrology, Tring, UK) and post-processed using VG Studio MAX v 3.0 (Heidelberg, Germany).

### Scanning electron microscopy

The samples were fixed in 4% formaldehyde/2.5% glutaraldehyde (SIGMA-ALDRICH) in 0.1 M Phosphate Buffer (PB) pH 7.4 for 5 h at RT. Samples were then washed in 0.1 M PB, cut in ~5 mm and cryoprotected in 25% sucrose, 10% glycerol in 0.05 M PB overnight. Samples were then fast frozen and fractured in liquid nitrogen using a stainless-steel probe and then placed back into the cryoprotectant at RT to thaw. Samples were stained in 1% OsO_4_/1.5% potassium ferricyanide, washed in H2O and dehydrated in a graded ethanol series, critical point dried with CO_2_ using a Leica EM CPD300 and mounted on aluminium stubs using adhesive carbon tabs. The samples were mounted to present the fractured surfaces to the beam and coated with a thin layer of platinum using a Quorum Q150 R S sputter coater. Gross images of the samples were taken prior to SEM imaging on a Leica M205C stereomicroscope. SEM images were recorded with a FEI Quanta 250 FEG scanning electron microscope.

### DNA quantification

DNA from native rat thymi and decellularised organs was extracted with the PureLink Genomic DNA MiniKit (Invitrogen) according to the manufacturer’s instructions. DNA samples were measured spectrophotometrically (NanoDrop™ 1000, ThermoFisher).

### Repopulation and in vitro culture of thymic scaffolds

Cell suspension in cFAD medium of cultivated TEC and TIC (ratio 5:1) were injected (2–3 M cells in 100 μl/lobe) using Insulin Syringes (Terumo, 29.5 G) into decellularised rat thymi. Stromal cells used for repopulation of scaffolds were derived from 4 months and 6 months old donors. Seeded thymic scaffolds were cultured for 5 or 6 days in cFAD medium and then injected with TN (400,000) ± VeraVec (200,000) cells in 50 μl volume of co-culture medium (DMEM-1X (Gibco), 10% FBS (SIGMA-ALDRICH), 1% Penicillin/Streptomycin (100X, SIGMA-ALDRICH), Triodothyronine (T3) (2 × 10^−9^ M SIGMA-ALDRICH), Insulin (5 µg/ml, SIGMA-ALDRICH) and Cytokines (Interleukin-7, 5 ng/mL (Invitrogen), Stem Cell Factor 5 ng/mL (Cell Signalling) and FLT3-L (5 ng/mL, CellGS)). Scaffolds were reconstituted only with TEC (*n* = 4), only TIC (*n* = 2) or with TEC and TIC (*n* = 5). Fully reconstituted scaffolds (with TEC, TIC and TN) were kept in co-culture medium (without cytokines, *n* = 3) and cultured up to 14 days in cFAD medium.

### Bone marrow reconstitution and subcutaneous transplantation

NSG (8–12-week-old) mice were sub-lethally irradiated with 3.75 Gy from a ^137^Caesium source (IBL 637 Gamma Irradiator). For primary engraftment, purified CD34^+^ cells (100 K CB-CD34^+^ or 200 K FL-CD34^+^/cells) were injected intravenously per mouse. Engraftment of human cells in the murine bone marrow was assessed at killing. Subcutaneous implantation of the scaffold was performed in NSG mice 4–6 weeks post-CD34^+^ injection. Mice were anaesthetised with a 5-2% isoflurane oxygen gas combination for induction and maintenance. Buprenorphine 0.1 mg Kg^−1^ was administered at the induction for analgesia. Under aseptic conditions, 1–3 midline incisions (0.4 cm) were performed on the back of the mice and scaffolds were inserted in lateral pockets^40^. Mice were culled at different time points ranging from 1 to 22 wpt.

We carried out subcutaneous transplantation in three independent experiments on a total of 16 NSG mice, 14 of which had been humanised with CB-CD34 cells and 2 with FL-CD34 cells: all mice showed bone marrow reconstitution. The mice were implanted with 37 repopulated scaffolds, of which 34 were retrieved. Specifically, eight mice were implanted with repopulated scaffolds containing TIC, TEC, VeraVec and CD34^+^ cells (*n* = 18); six mice received repopulated scaffolds without CD34^+^ cells (*n* = 13), other two mice received scaffold without VeraVec cells (*n* = 6). In addition, two mice received empty (*n* = 2) and 2 mice TIC only scaffolds (*n* = 6). No significant alterations in the extent of blood vessels correlated with the absence of endothelial cells in the scaffolds.

The NSG-nude female mice (8–12-week-old) were sub-lethally irradiated with 3.25 Gy and subsequently injected intravenously with purified human CD34^+^ cells. NSG-nude mice had a lower rate of humanisation than hairy littermates as established by bone marrow (BM) engraftment (4 out of 12 mice showed 27-55% hCD45^+^ cells against the 50–70% hCD45^+^ in 5 out 5 hairy littermates). Mice with no BM reconstitution were excluded from further analysis. BM and spleen were retrieved from humanised mice (one at 10 wpt and one at 18 wpt) engrafted with stroma-repopulated scaffolds and processed for flow cytometry analysis or sorting. Two humanised NSG-nude mice grafted with empty scaffolds showed no peripheral repopulation (no hCD3^+^ cells detectable in the spleen) at 18 wpt and 20 wpt.

### Scaffold FACS analysis and sorting

Repopulated natural scaffolds were mechanically and enzymatically dissociated using Collagenase I (2 mg/mL, SIGMA, C0130), Dispase II (1 U/mL, Roche) and DNAse I (80 μg/mL, Roche) in RPMI 1640 and 2% FBS. At the end of digestion, cell suspension was passed through a cell strainer (100 μm) and then stained with antibodies for FACS sorting or analysis.

We performed sorting and/or analysis of a total of 16 Scaffolds of which 9 were repopulated with CD34^+^ HSC and 7 without CD34^+^ HSC. Four non-repopulated controls and two scaffolds with only TIC, CD34^+^ and VeraVec were harvested at 22 wpt and 11 wpt, respectively. Scaffolds were analysed and no thymocytes were detected. We found CD4/CD8 SP and DP in 14 repopulated scaffolds: in 7 out of 9 scaffolds repopulated with CD34^+^ HSC and in 7 out of 7 scaffolds repopulated without CD34^+^ HSC.

### Statistical analysis

For all the experiments included in this study, three or more biological replicates were performed; the exact sample size (*n*) for each experimental group/condition, given as a discrete number. Statistical analysis was performed using two-way ANOVA non-parametric, unless stated otherwise. Plots and graphs were generated with GraphPad PRISM 8.

### Reporting summary

Further information on research design is available in the Nature Research Reporting Summary linked to this article.

## Supplementary information

Supplementary Information

Description of Additional Supplementary Files

Supplementary Movie 1

Supplementary Movie 2

Reporting Summary

## Data Availability

RNA-sequencing data are deposited at ArrayExpress database under accession number E-MTAB-9641 and scRNA-sequencing at Gene Expression Omnibus (GEO) database under accession number GSE159745. All datasets generated and/or analysed during the current study are available from the corresponding author upon reasonable request.
