# Supplementary material for: Reconstitution of a functional human thymus by postnatal stromal progenitor cells and natural whole-organ scaffolds
Source: Nat Commun. 2020 Dec 11;11:6372. doi: 10.1038/s41467-020-20082-7 (PMC7732825; doi:10.1038/s41467-020-20082-7)
Supplement: Supplementary file 1 — Supplementary Information [file 41467_2020_20082_MOESM1_ESM.pdf]

Supplementary Information

Reconstitution of a functional human thymus by postnatal stromal progenitor cells and natural whole-organ scaffolds

Campinoti, Gjinovci, Ragazzini et al.

- Supplementary Figures 1-10
- Supplementary Tables 1-3

Supplementary Fig. 1

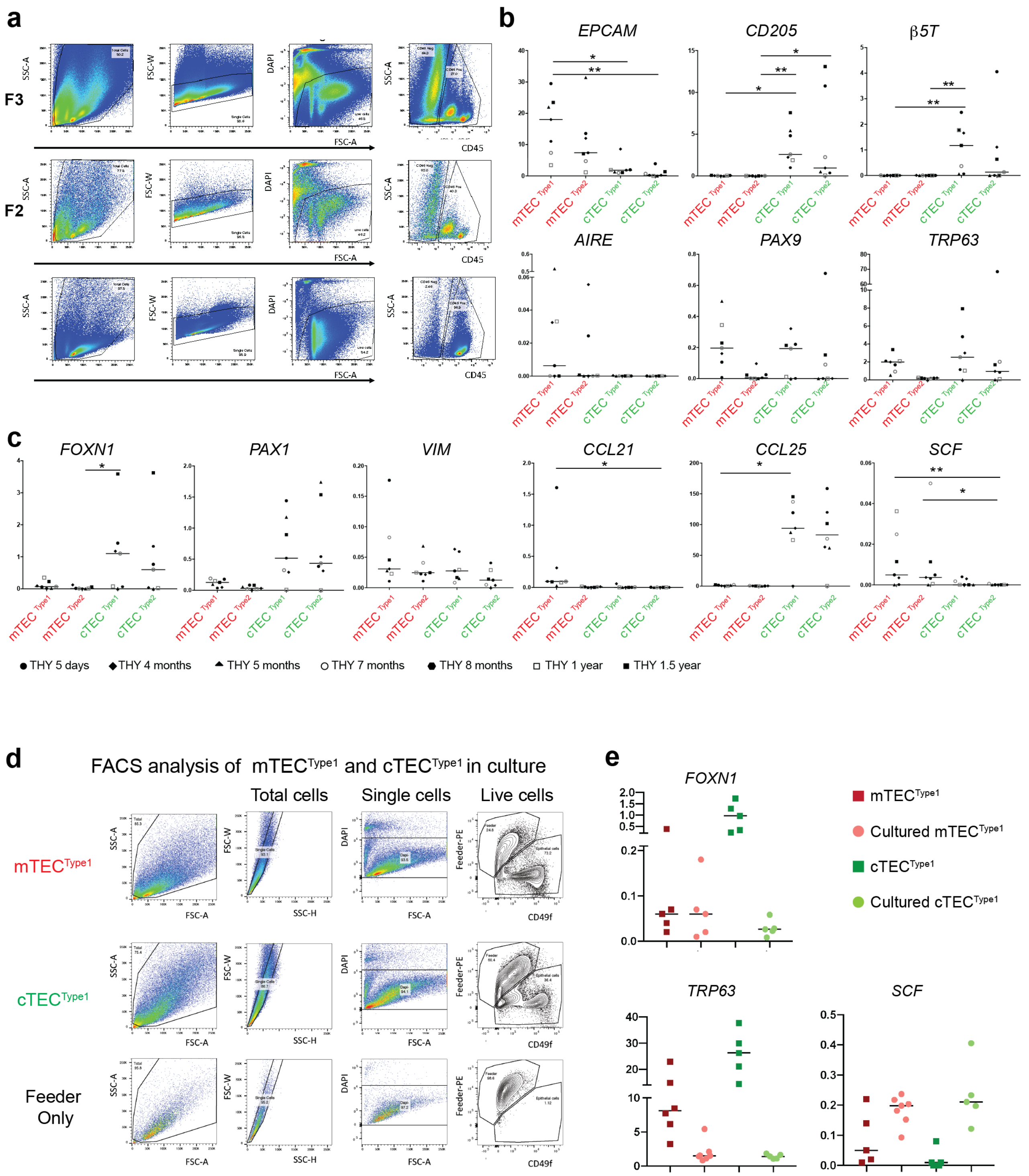

Supplementary Fig. 1 | Phenotypic analysis of sorted thymic epithelial cells. a, FACS plots representing analysis of the progressive enrichment fractions (F2-F3) of $CD45^-$ population before sorting TEC and TIC subpopulations; $CD45^-$ (PE^-) population is about 2% after just several washing of the total dissociated postnatal thymus and increases up to 64% after 3 enrichment steps; n=18. **b-c,** RT-qPCR analysis of freshly sorted cTEC and mTEC (Type1 and Type2) from 7 thymi (donors age, 5 days-1.5 years old). *EPCAM* and *CD205* transcripts expression confirmed the purity of the sorting strategy, together with detection of cortical marker *b5T* only in cTEC and *AIRE* only in mTEC. *Vimentin* transcripts are detected in all freshly isolated cTEC and mTEC, while transcription factors (*PAX1, FOXN1, PAX9* and *TRP63*) and chemokines (*CCL21, CCL25, SCF*) were differentially expressed among the freshly isolated subpopulations (n=7 independent thymi; bars indicate the mean value). Relative gene expression to enriched CD45-negative cells is shown in Y axis; significance: two-way ANOVA, non-parametric; *p<0.05; **p<0.01. **d,** Representative FACS analysis of expanding (passage 5) mTEC Type1 and cTEC Type1 demonstrates CD49F-AF488 expression in TEC which are negative for anti-feeder-PE that label 3T3 feeder cells; n=4. **e**, RT-qPCR analysis showing gene expression levels for *FOXN1, TRP63* and *SCF* of freshly-isolated mTEC Type1 and cTEC Type1 *versus* cultured counterparts. mRNA relative abundance values normalised to *HPRT* are shown in Y axis. Donors age from 3 days to 1.5-year-old (n=5; mean ± SE).

Supplementary Fig. 2

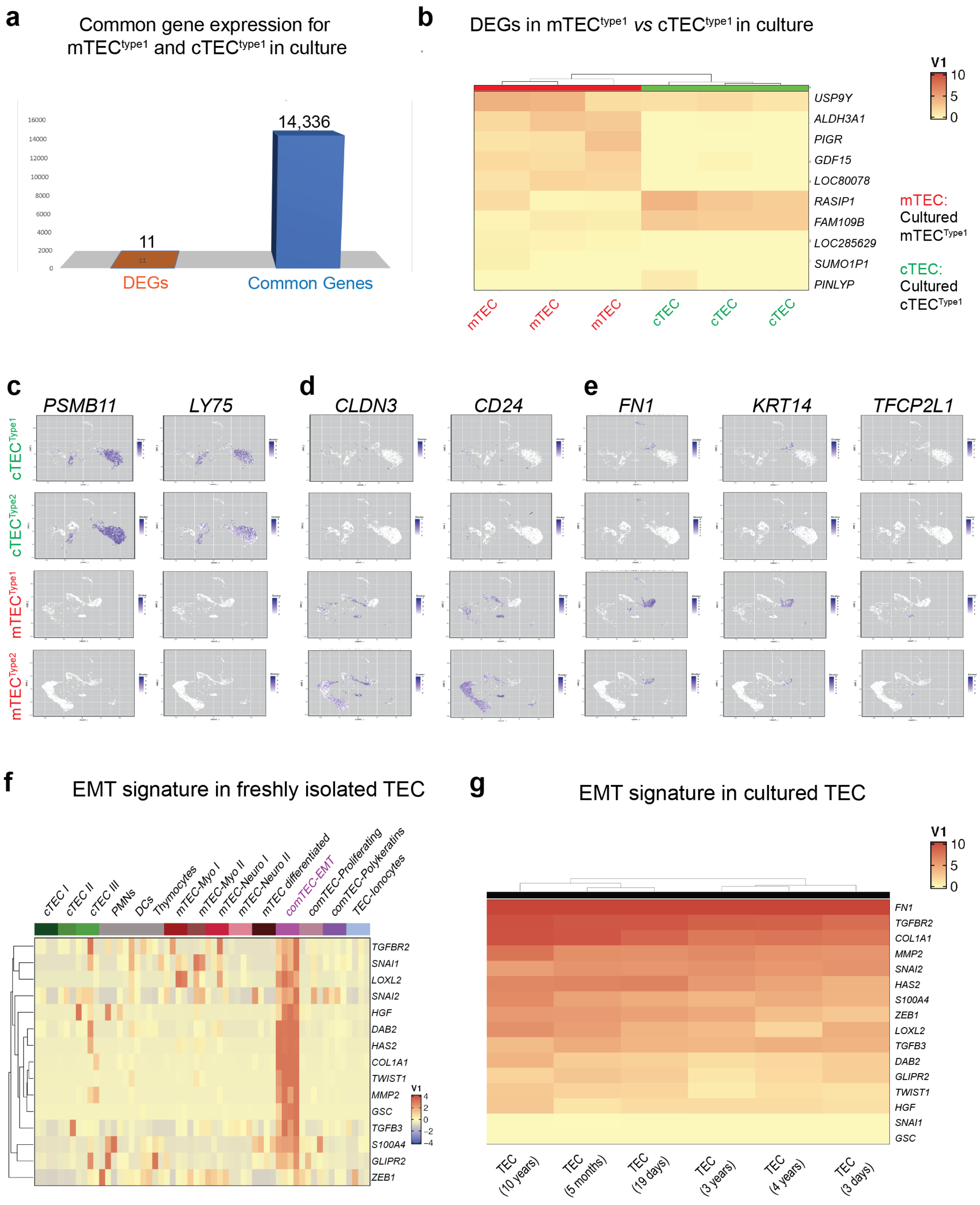

Supplementary Fig. 2 | Transcriptional profiling of freshly-isolated and cultured thymic epithelial cells. **a, b** Gene expression signature of cultivated cTEC and mTEC. Heatmap and hierarchical clustering of samples based on the most differentially expressed genes (DEGs): only 11 genes out of 14347 are differentially expressed between cultured mTEC and cTEC (=0.08%). Significant DEGs were selected applying a 0.05 cut-off on FDR and a threshold of 1.5 on logFC. Area under the curve (AUC) summary intensity plots of single genes expressed in cTEC: *PSMB11, LY75* (**c**); mTEC: *CD24, CLDN3* (**d**) and comTEC: *FN1, KRT14 and TFCP2L1*(**e**); single-cell expression values are represented as calculated median intensity of area under the curve, purple scale. **f**, Heatmap showing EMT genes expression in freshly isolated single-cells from human postnatal thymic stroma (EMT cluster). TEC clusters are listed on the x axis, genes are listed on the y axis **g**, Heatmap of *in vitro* expanded TEC (passage IV) showing expression of mesenchymal genes. Entries display average log-normalized read counts per million for n=6 biological replicates of TEC cultures (donor age: 3 days to 10 years old).

Supplementary Fig. 3

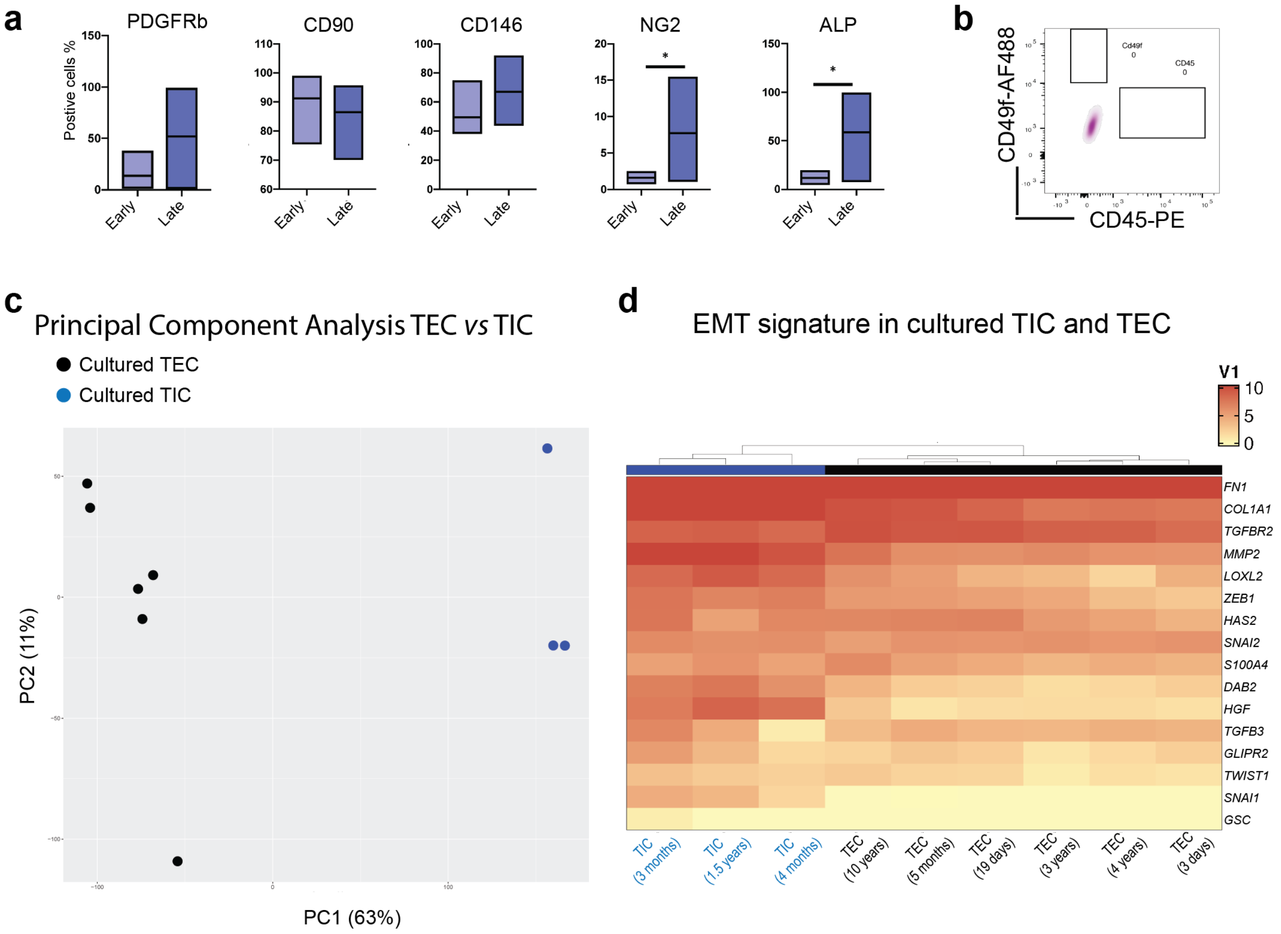

Supplementary Fig. 3 | Cultivated thymic interstitial cells are distinct from thymic epithelium. **a**, Floating bars (minimum to maximum value with line at the median) of FACS analysis showing percentage of PDGFRβ (PE), CD90 (AF700), CD146 (BV711), NG2 (AF488) and ALP (APC) positive TIC at early (II-III) and late (IV-VII) passages by FACS analysis (n=4; median ± SE; significance: two-way ANOVA, non-parametric; *p=0.035 for NG2 and p=0.014 for AP). **b**, Representative FACS analysis of expanding TIC demonstrates absence of both epithelial (CD49F^{+}) and CD45^{+} cells at early (II) passage (N=3). **c**, PCA plot of cultivated TEC *vs* TIC in culture showing sample variance according to PC1. (63%) and PC2 (11%) **d**, Heatmap of *in vitro* expanded TEC vs TIC showing expression of genes related to Epithelial Mesenchymal Transition (EMT). Entries display average log-normalized read counts per million for n=3 TIC and n=6 TEC biological replicates (donor age: 3 days to 10 years old).

Supplementary Fig. 4

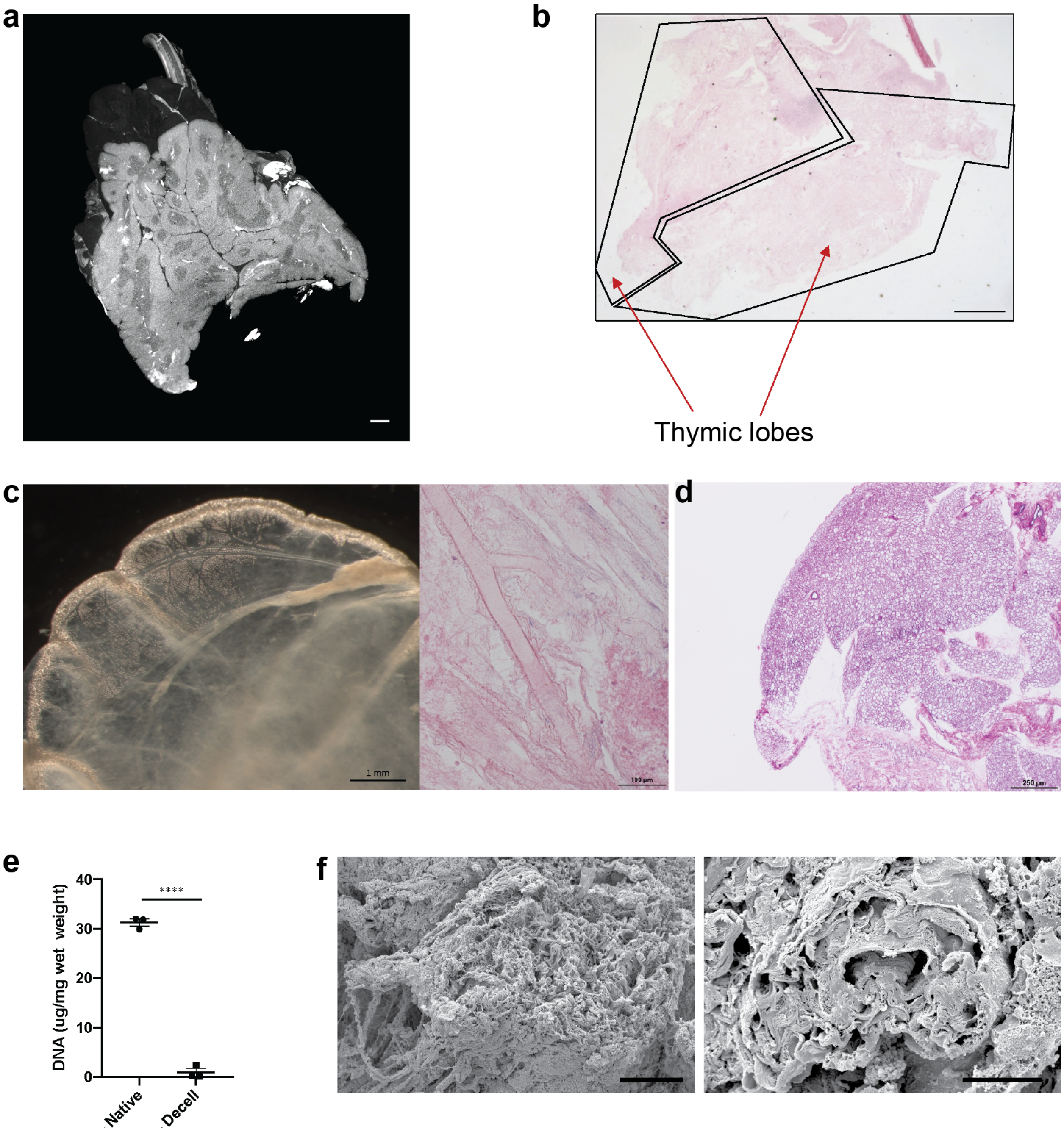

Supplementary Fig. 4 | Advanced imaging of whole organ rat thymus scaffolds. a, Segmentation of a micro-CT image of cannulated whole thymus organ shows organisation of cortex and medulla areas within both lobes, does providing a virtual histology of a whole organ. Scale bar, 1.2 mm. **b,** Haematoxylin&Eosin (H&E) of a rat thymus scaffold demonstrates homogenous decellularisation of the entire two thymic lobes; n=3 scaffolds. Scale bar, 500 µm. **c,** Gross microscopy (left panel) and H&E of a decellularised thymus scaffold showing preservation of vasculature wall; n=3 scaffolds. Scale bar, 1 mm (left panel); 100 µm (right panel). **d,** Van Gilsen's stain of a paraffin section of a decellularised rat thymus scaffold showing preservation of elastin fibres; n=3 scaffolds. Scale bar, 250 µm. **e**, DNA quantification of fresh thymus tissue (Native) and decellularised thymus scaffold (Decell) demonstrates removal of DNA in the acellular scaffold (n=3 scaffolds, data presented as mean value ± SD); significance: unpaired t-test two-tailed, ****$p<0.0001$. **f**, Scanning Electron Microscopy (SEM) of decellularised scaffold shows extra-cellular matrix (ECM) fibre organisation at high resolution. Scale bar, 50 µm (left panel); 10 µm (right panel); n=2 scaffolds.

Supplementary Fig. 5

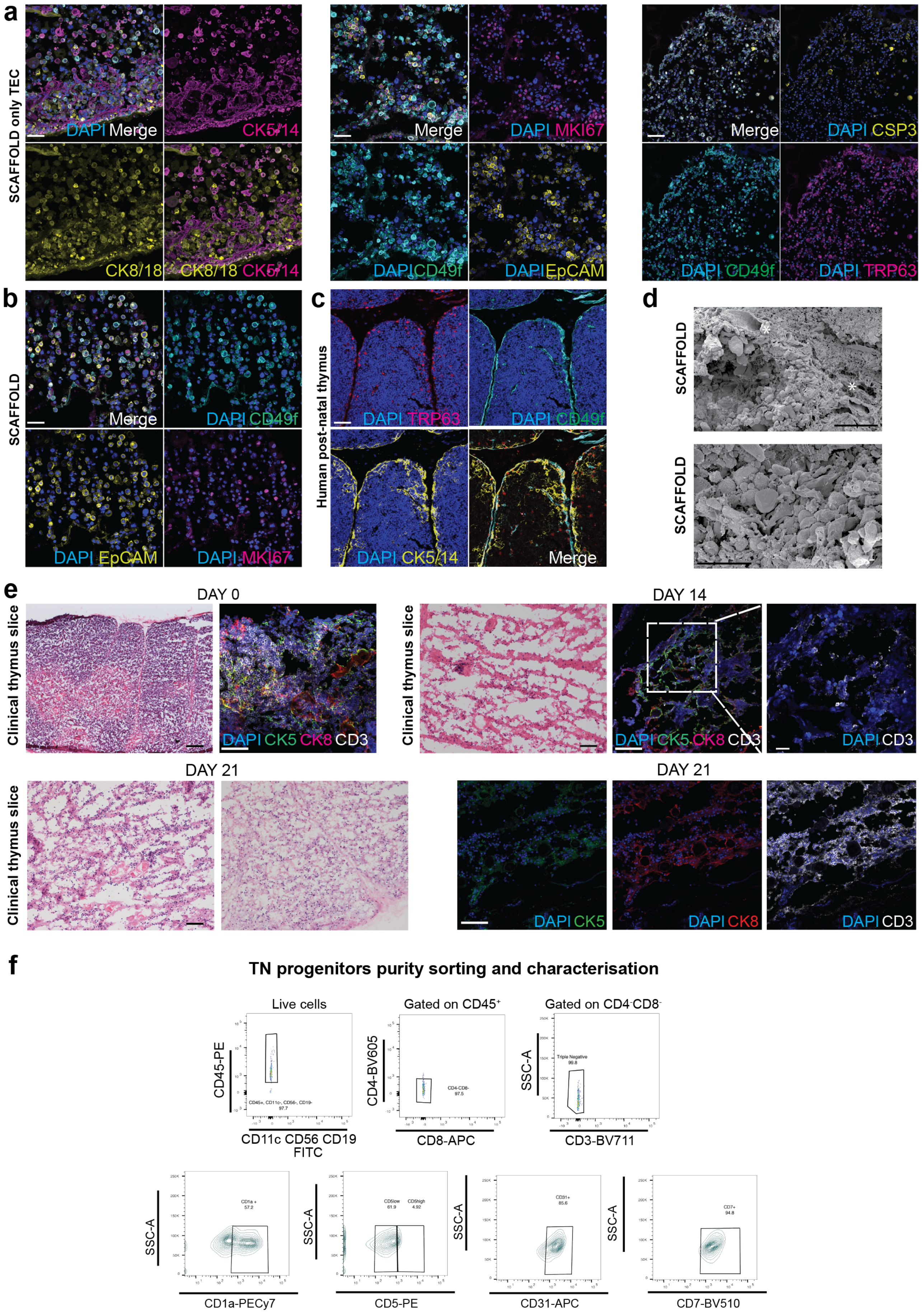

Supplementary Fig. 5 | Functional repopulation of whole organ thymus scaffolds and comparison with clinical thymus slices. a, Immunofluorescence labelling of thymic epithelial cells (TEC) seeded and grown within a decellularised scaffold demonstrating viable (Caspase3-negative), proliferative ($Ki67^+$), $CK5^+CK8^+$ double and single positive TEC; n=4 repopulated scaffolds. Scale bar, 50 µm. **b,** Immunohistochemistry of decellularised thymus scaffold repopulated with both TEC and TIC demonstrating presence of proliferative ($Ki67^+$) $EpCAM^+$ and $CD49f^+$ cells upon culture; n=4 repopulated scaffolds. Scale bar, 50 µm. **c,** Immunofluorescence of a postnatal human thymus showing expression of TPR63 mainly in subcapsular regions where TEC are $CK5/14^+$ and $CD49f^{high}$; n=5 thymi. Scale bar, 50 µm. **d,** Scanning Electron Microscopy (SEM) images of a scaffold repopulated with TEC and TIC and cultured for 5 days and re-organised around an intact vascular structure (indicated with asterisks, *); n=2 repopulated scaffolds. Scale bar, 100 µm. **e**, Haematoxylin&Eosin (H&E) staining of a freshly prepared (DAY 0) clinical thymus slice and immunofluorescence against CK5 (green), CK8 (red) and CD3 (white) - upper left panels; histology and immunohistochemistry of clinical thymus slices cultivated for 14 and 21 days as prepared for transplantation into athymic patients: cyto-architecture is disrupted and $CD3^+$ (white) cells are still detectable; nuclei counterstained with DAPI (blue); n=3 sliced thymi. Scale bar, 50 µm. **f**, Upper panels: FACS purity check of sorted Triple Negative lymphoid progenitors (TN, $CD3^-CD4^-CD8^-$), n=15; lower panels: representative FACS analysis for the expression of CD1a (PE-Cy7), CD5 (PE), CD7 (BV510) and CD31 (APC) of sorted TN cells, n=3.

.

Supplementary Fig. 6

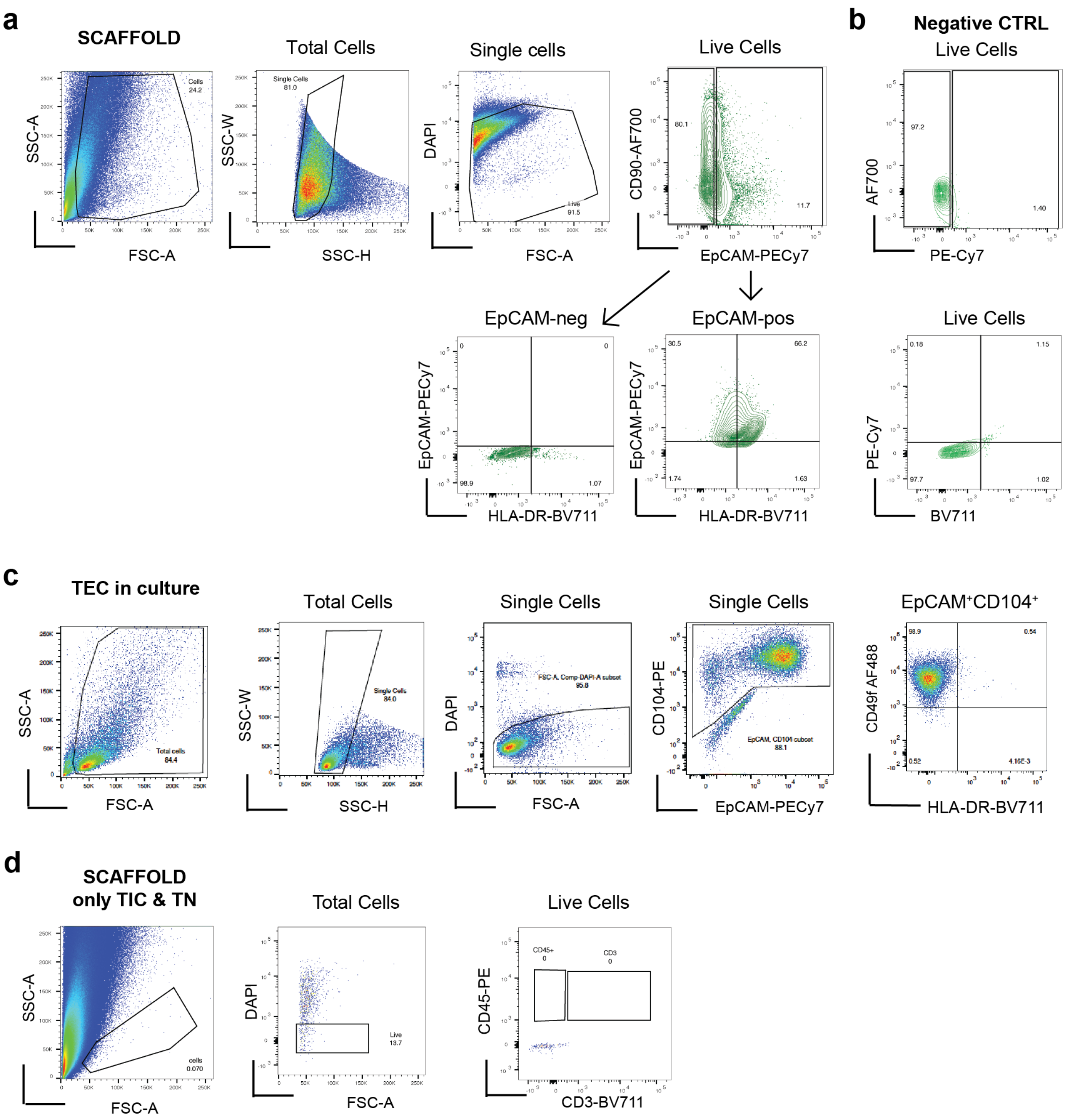

Supplementary Fig. 6 | Thymus scaffolds *in vitro,* negative control and stromal compartment analysis. a, FACS plots showing gating strategy and analysis of epithelial cells co-cultured with TIC and TN *in vitro* for 8 days. HLA-DR is expressed by $EpCAM^+$ cells but not by $EpCAM^-$ cells (Live cells: 70000 events; n=3 repopulated scaffolds)**. b**, Unstained control for analysis in panel a. **c,** FACS plots representing the gating strategy and analysis of cultivated TEC (passage 4) positive for CD104 (Integrin β4), EpCAM, CD49f but negative for HLA-DR; n=5 independent cultures. **d,** FACS analysis of *in vitro* scaffold co-cultured with TIC and TN shows no $CD45^+$ live cells within the scaffold after 8 days of culture (Live cells: 161 events; n=2).

Supplementary Fig. 7

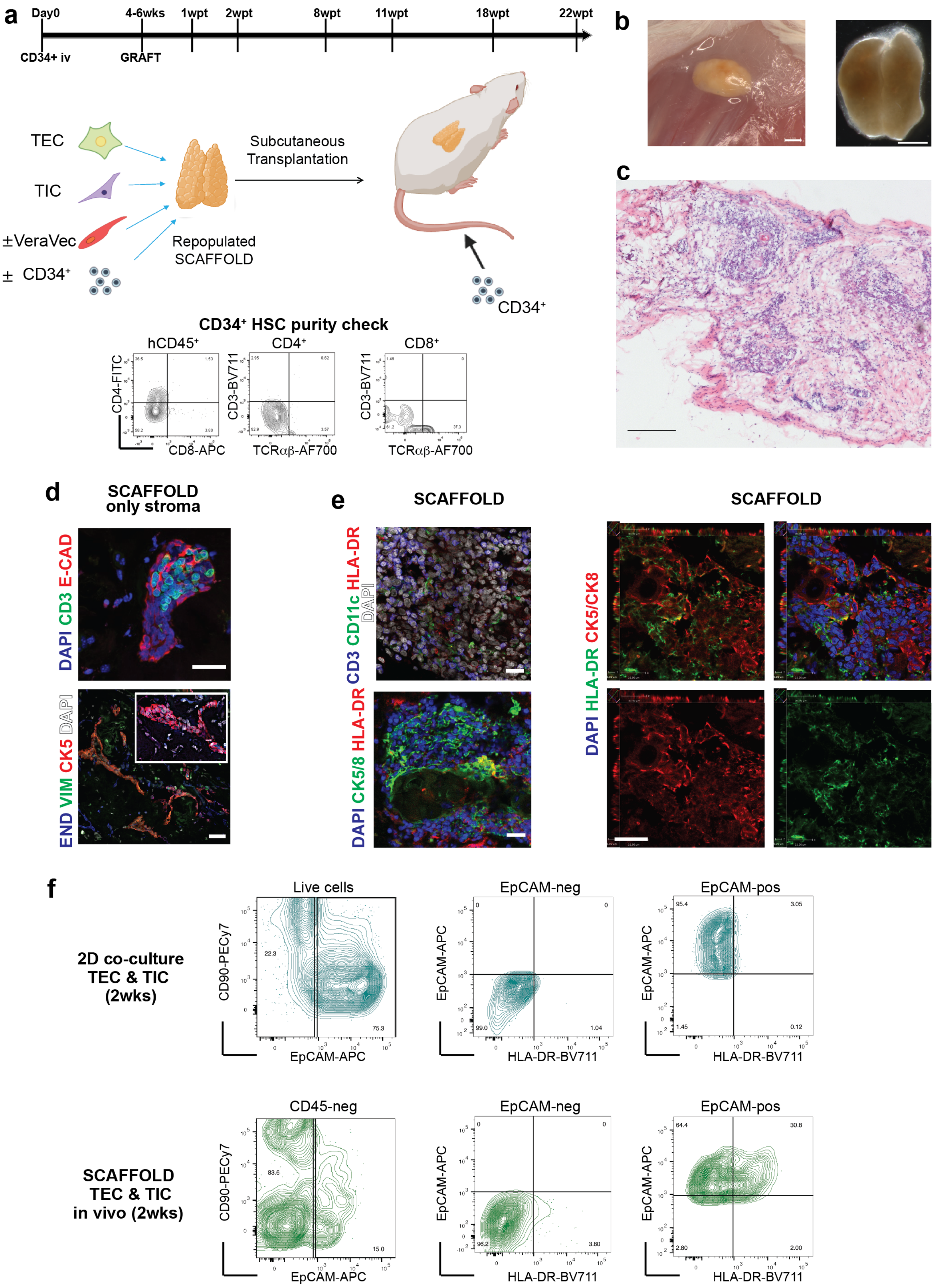

Supplementary Fig. 7 | Whole organ thymus scaffolds mature *in vivo*. a, Schematic summarising the *in vivo* transplantation assay. Humanisation of immunodeficient mice occurred 4 to 6 weeks prior subcutaneous grafting of repopulated scaffolds which were then harvested at different time points: 1, 2, 8, 11, 18 and 22 weeks post-transplant (wpt). The schematic was created with BioRender.com. Bottom panels: FACS plots showing purity check of $CD34^+$ HSC used for scaffold repopulation and mouse humanisation, n=6. **b**, Gross microscopy appearance of a representative reconstituted Scaffold 11 wpt before (left) and after (right) harvesting. Harvested scaffold has been cut in two parts: one half was processed for FACS analysis or sorting, the other half was processed for histology. Scale bar, 1 mm. **c**, Representative H&E of a repopulated scaffold at 11wpt showing many areas of thymic reorganisation with both stroma and thymocytes within remodelled ECM. n=4 scaffolds in two independent experiments. Scale bar, 250 μm. **d**, Immunohistochemistry of thymus scaffold repopulated with only stroma demonstrates compact epithelial ($CK5^+$, red) areas and initial migration and differentiation of HSCs towards $CD3^+$ cells (green) at 11 wpt; n=4 scaffolds. Scale bar, 40 μm. **e**, Immunofluorescence (18wpt) demonstrating presence of HLA-DR-positive epithelial ($EpCAM^+$) and dendritic ($CD11c^+$) cells interacting with $CD3^+$ cells. Scale bar, 20 μm. Nuclei counterstained with DAPI (left top panel: white; left bottom panel: blue). Right panel: High magnification of a single focal plane showing co-expression of HLA-DR in green with CK5-8 in Red; n=4 scaffolds in two independent experiments. Scale bar, 25 μm. **f**, FACS analysis of 2D co-cultured TEC and TIC (upper panels, live cells= 12000 events) and fully 3D repopulated scaffold (TEC, TIC, VeraVEC and $CD34^+$) harvested at 2wpt (bottom panels: live cells, $CD45^-$=2100); n=2 independent experiments. HLA-DR profile is shown on EpCAM negative and positive populations.

Supplementary Fig. 8

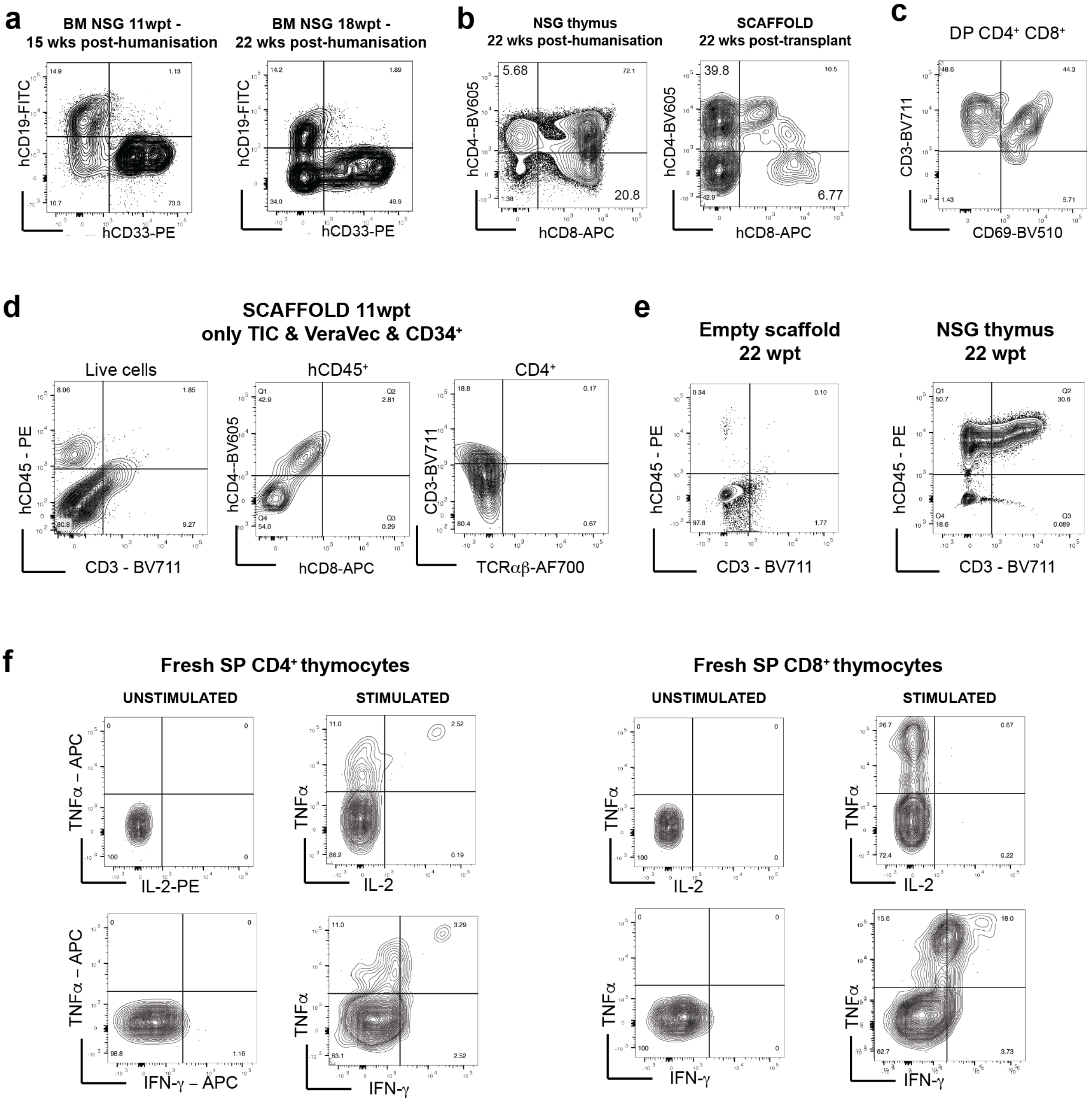

Supplementary Fig. 8 | Thymus scaffolds support *in vivo* T cell development. a, FACS analysis of bone marrow (BM) of a humanised NSG mice at 15 weeks post-humanisation (11 wpt) and 22 weeks post-humanisation (18 wpt) demonstrate $CD34^+$ HSC ability to differentiate towards myeloid ($CD33^+$) and B cell ($CD19^+$) lineage (n=16, live cells=50000). **b**, FACS analysis of $hCD45^+$ population recovered following dissociation to single cells of NSG mouse thymus 22 weeks after humanisation (left panel, live cells=300000) and of a repopulated thymus scaffold 22 wpt (right panel, live cells=5000): CD4/CD8 ratio is higher when the instructing stroma is of human origin (n=2). **c**, FACS analysis of repopulated scaffold at 11wpt showing CD69 expression by DP thymocytes (same scaffold as in Fig. 6g, live cells=1100; n=2). **d**, FACS analysis of *in vivo* repopulated scaffold with TIC, VeraVEC and $CD34^+$ HSC at 11 wpt (n=2, live cells= 4800), showing few $CD3^-$ $hCD45^+$ events (left panel), absence of mature T cells (mid and right panels). **e**, Absence of $hCD3^+$ cells among live cells in a transplanted empty scaffold (n=4) at 22wpt (left panel) compared to endogenous thymus of the same mouse (right panel). **f**, Representative FACS analysis of human SP $CD4^+$ and $CD8^+$ thymocytes freshly isolated from a paediatric thymus (2 years old) and stimulated with PMA-*Ionomycin* demonstrating that human SP thymocytes produce only limited amount or none of IL2. CD8 SP cells, produce the highest level of IFNγ and TNFα similarly to what we observed in thymocytes developed within the human scaffolds (n=3, live cells=6500).

Supplementary Fig. 9

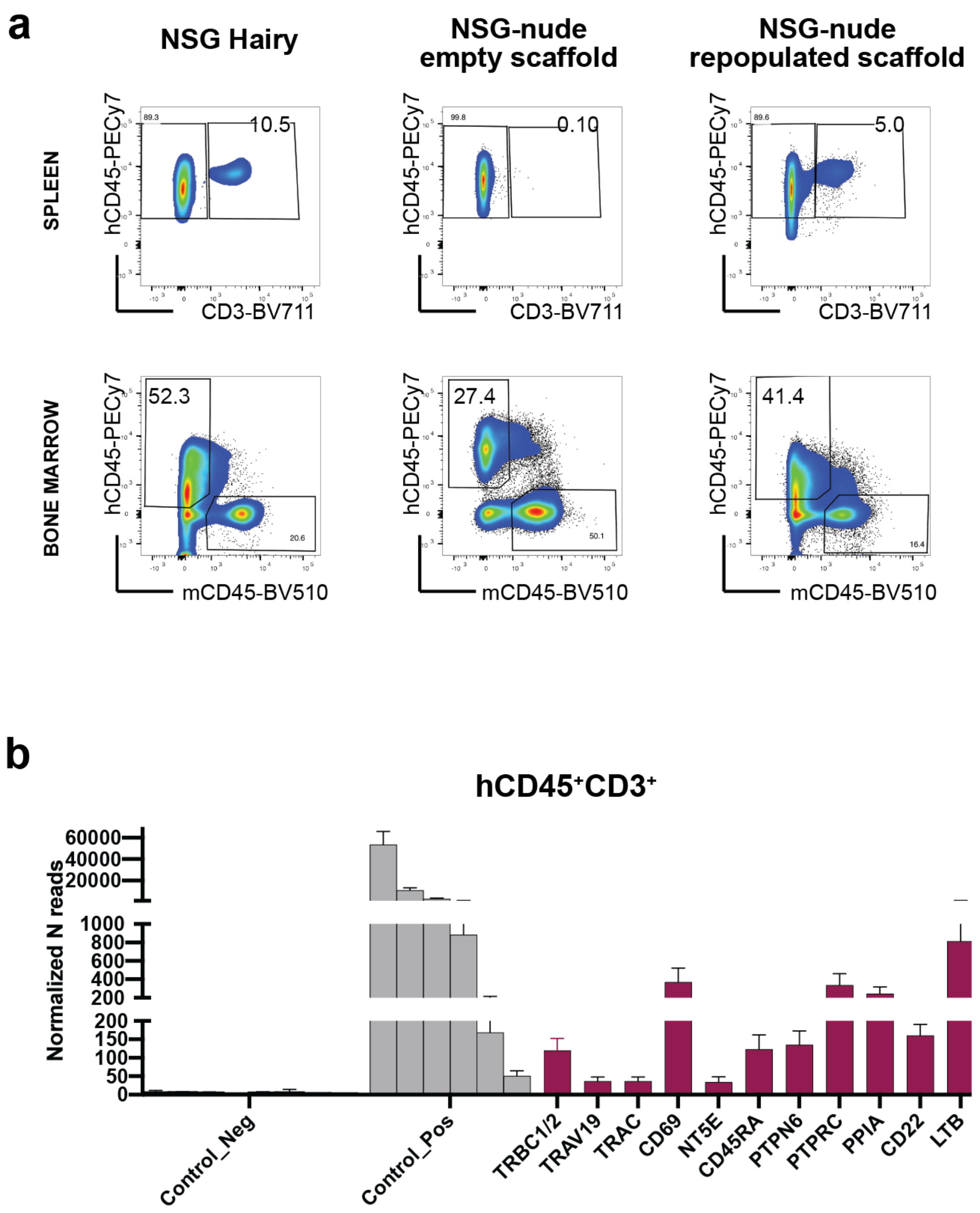

Supplementary Fig. 9 | Peripheral repopulation by T cells in athymic NSG-nude mice. a. FACS plots showing hCD45$^+$ population (gated on at least 20,000 live cells) of spleen and bone marrow of humanised NSG mouse (Hairy, left panel; n=5 mice), humanised NSG-nude mouse grafted with empty scaffolds (middle panel; n=2 mice) and humanised NSG-nude mouse grafted with repopulated scaffolds (right panel; n=2 mice). **b.** NanoString analysis was performed on hCD45$^+$CD3$^+$ cells sorted from humanised NSG-nude spleen (n=2, 1869 and 8081 sorted events) using commercially available NanoString nCounter™CAR-T Characterisation panel. Representative TCR signalling signature genes expressed by hCD45$^+$CD3$^+$, i.e. *TRB1/2, TRAV19, TRAC, CD69, NT5E, CD45R, PTPN6, PTPRC, PPIA, CD22* and *LTB*. Analysis of positive and negative control are included.

Supplementary Fig. 10

a **Gating strategy: TIC IN CULTURE corresponding to analysis of Figure 3e and Suppl 3a**

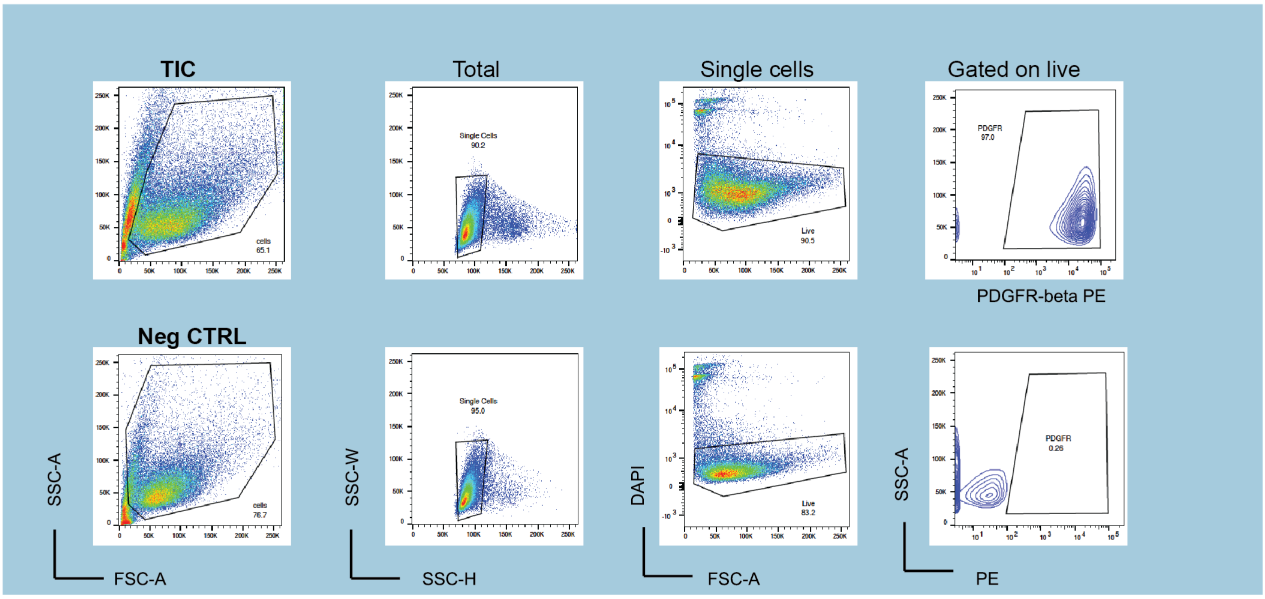

b **Gating strategy: TIC IN CULTURE corresponding to analysis of Suppl Figure 3b**

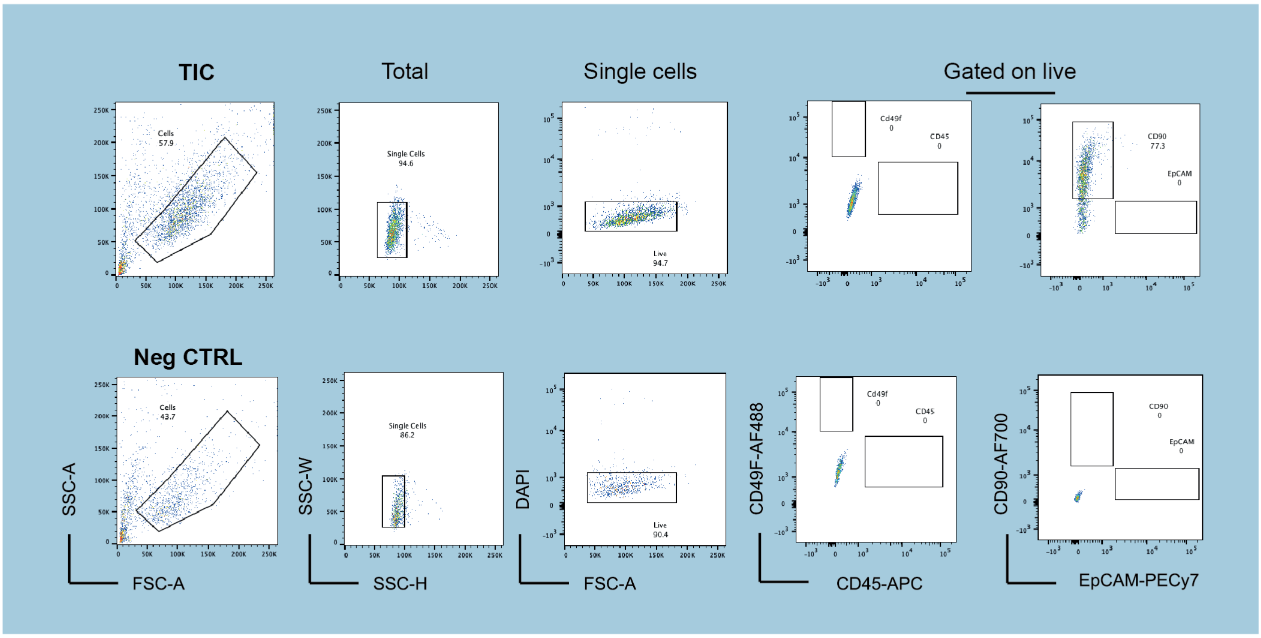

c **Gating strategy: TN purity check corresponding to Suppl Fig.7f top panel**

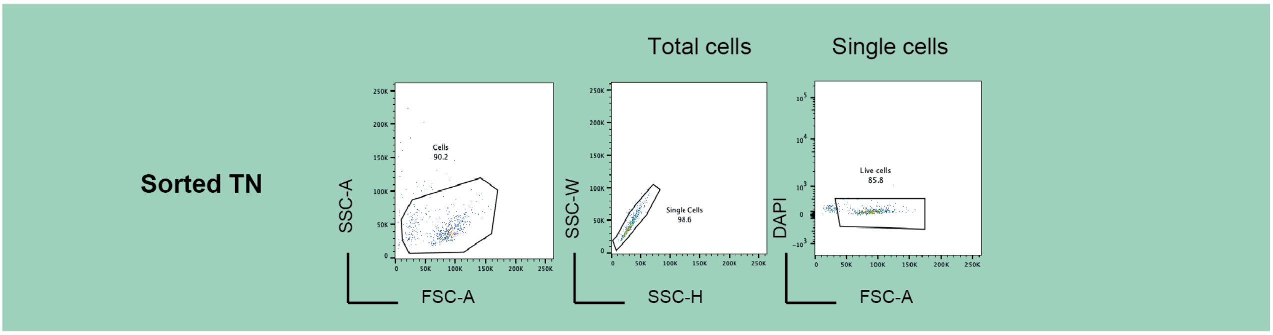

d **Gating strategy: Total thymocytes and TN corresponding to Suppl Fig. 7f bottom panel**

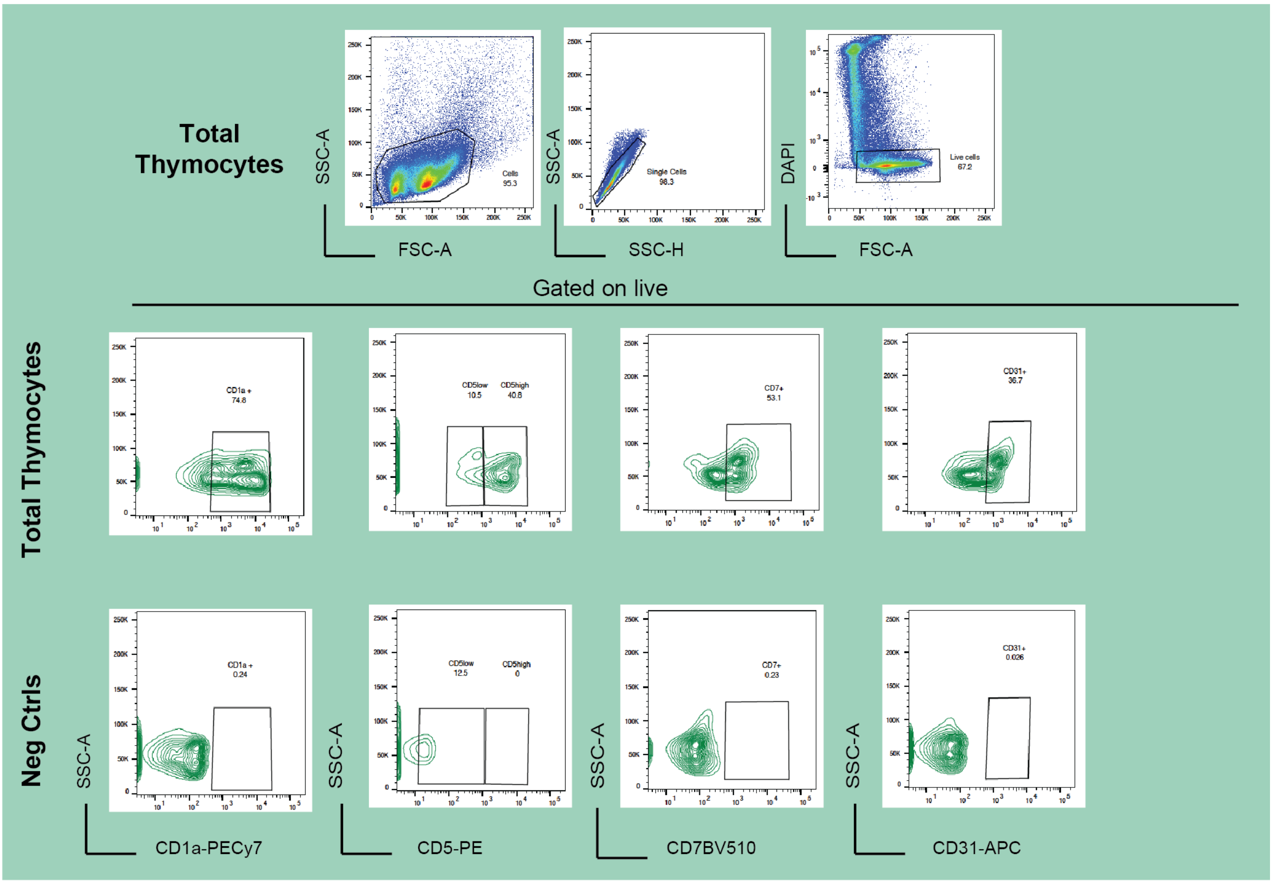

e **Gating strategy: In vivo scaffold analysis corresponding to Fig. 6f,g, Suppl Fig. 8b-e**

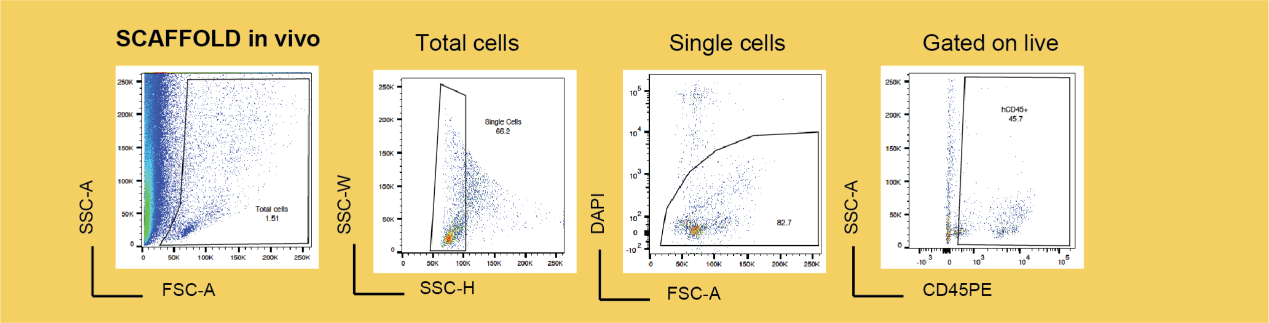

f **Gating strategy: PMA-Ionomycin assay corresponding to analysis in Fig.6h and Suppl Fig.8f**

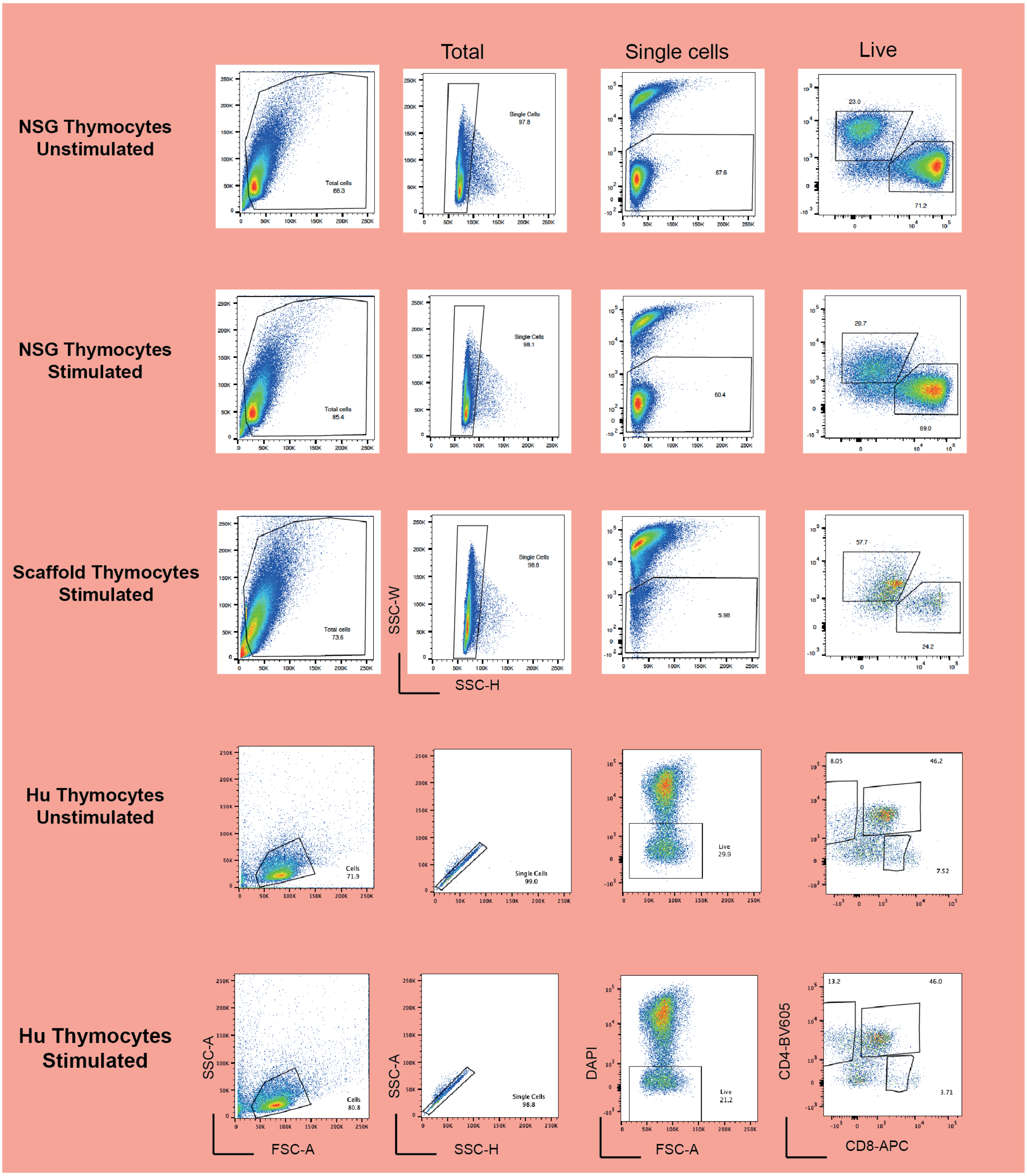

g **Gating strategy: purity of CB-HSC corresponding to Suppl Fig. 7a**

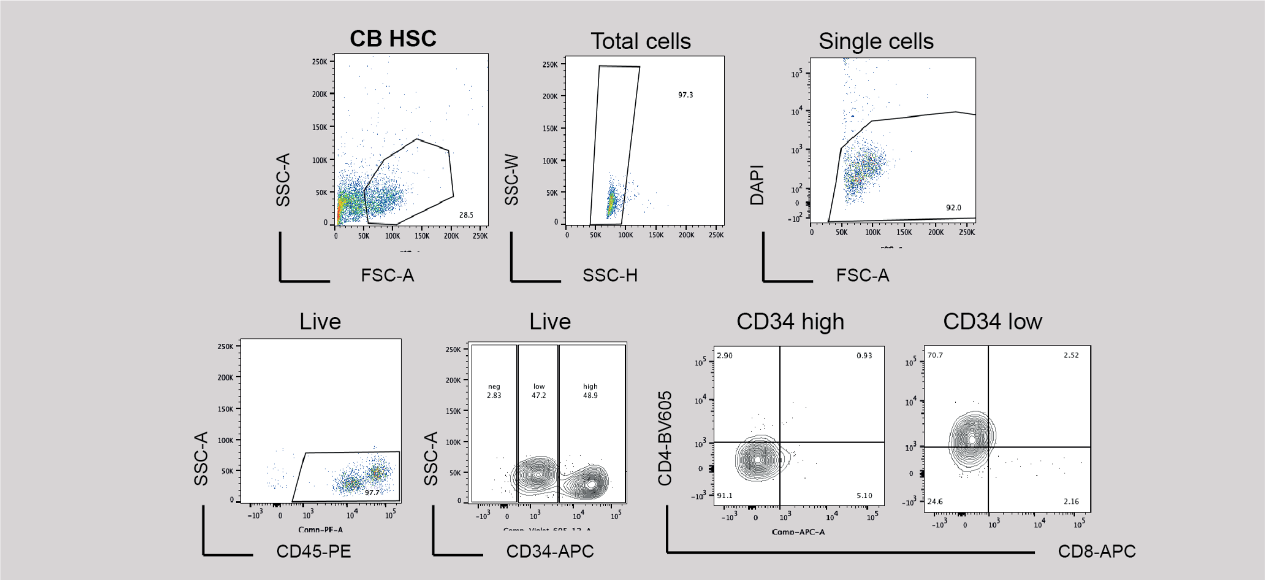

h **Gating strategy: Nude mice humanisation and perifery analysis corresponding to Suppl Fig. 9**

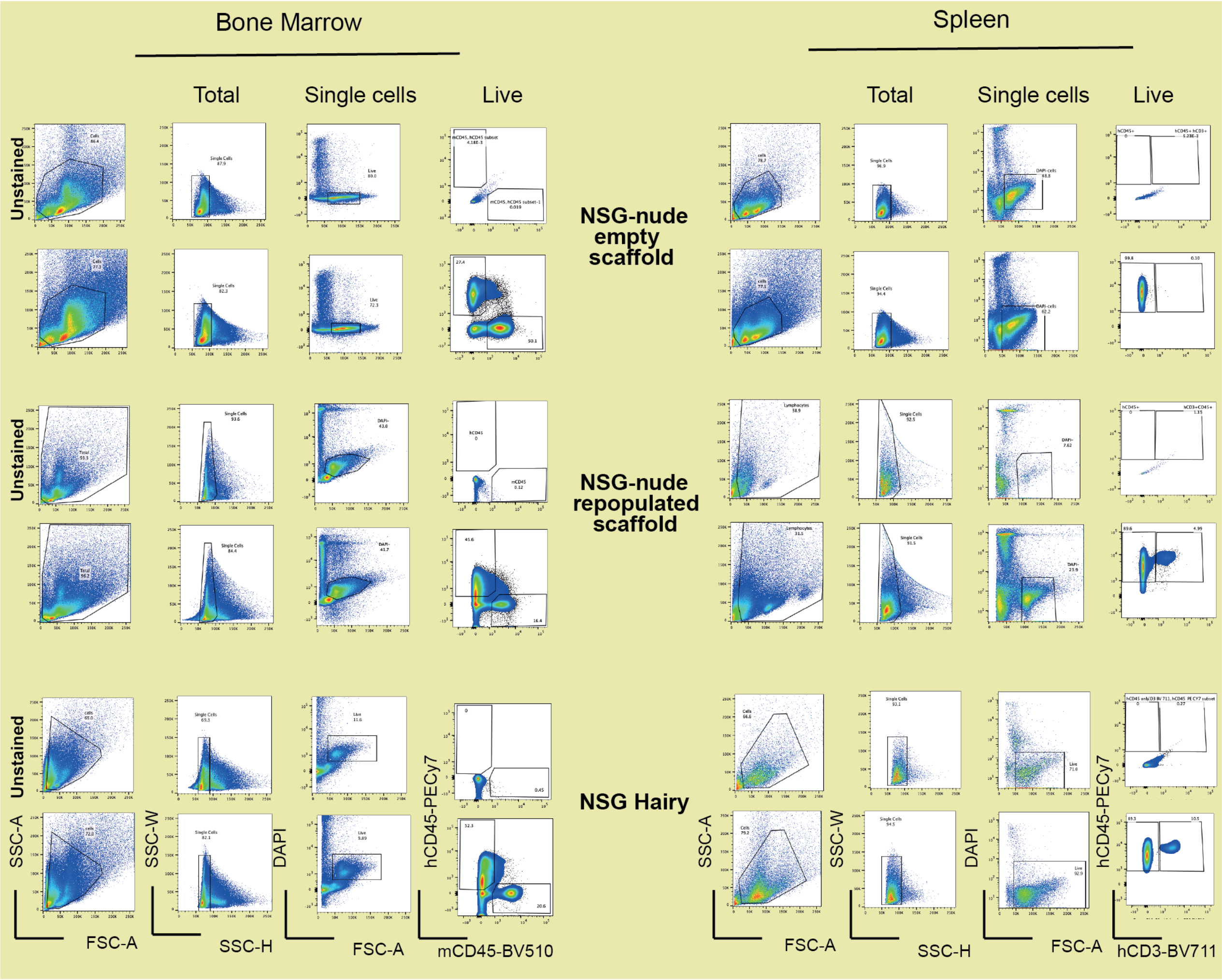

Supplementary Figure 10 | Gating strategies of flow cytometry analyses.

a,b Gating strategy used to examine by flow cytometry molecular markers expressed by human thymic interstitial cells. TIC have been gated sequentially for single-cells, live cells and markers expression. Unstained negative control is shown in the bottom panels. **a.** This gating strategy was used to examine relevant parameters in Figure 3e and quantify marker expression in Supplementary Figure 3a. **b.** This gating strategy was used to verify the absence of hCD45 and epithelial markers in TIC in Supplementary Figure 3b.
c. Gating strategy used to determine purity check of sorted triple negative thymocytes: total, single and live cells gates are displayed. Cells were ~85% viable after sorting. **d.** Gating strategies used to examine T cell markers in human triple negative (TN) and total thymocytes: total, single and live cells gates are displayed (top panel), total thymocytes (middle panel) and corresponding negative control (unstained, bottom panel). This gating strategy is relative to Supplementary Figure 7f, bottom panels.
e. Gating strategy used to examine thymocytes development within repopulated scaffolds transplanted subcutaneously in NSG mice and harvested at different time points. FACS panels display sequential gates for single cells, live cells and $hCD45^+$ gated on live cells. This gating strategy is relative to Figure 6f,g and Supplementary Figure 8b-e.
f. Gating strategy used to examine cytokine production of thymocytes after stimulation with PMA-*Ionomycin*. Sequential gates for single-cells, live cells and CD4 *vs* CD8 is displayed from the top to the bottom for NSG thymocytes unstimulated, stimulated, scaffold thymocytes stimulated, human thymocytes unstimulated and stimulated, respectively. This gating strategy is relative to Figure 6h and Supplementary Figure 8f.
g. Gating strategy used to determine purity check of human CB HSC used to humanise NSG and NSG-nude mice. Top panel displays sequential gates for single cells and live cells. Bottom panel shows CD45 (left plot), CD34 (middle plot) expression and CD4 & CD8 expression by high and low $CD34^+$ cell population (right plots). This gating strategy is relative to Supplementary Figure 7a FACS analysis.
h. Gating strategy used to analyse bone marrow reconstitution and spleen of NSG-nude mice. Sequential gates for single-cells, live cells and human *vs* mouse CD45 are displayed for NSG-nude empty scaffold, repopulated scaffolds and NSG Hairy mouse along with its unstained counterpart (left panels). Sequential gates for single-cells, live cells and human CD45 and CD3 are displayed for NSG Nude empty scaffold, repopulated scaffolds and NSG hairy mouse along with its unstained counterpart (right panels). This gating strategy is relative to Supplementary Figure 9a.

Supplementary Table 1 | List of antibodies used for flow cytometry

Antigen	Fluorocrome	Dilution	Clone	Reactivity	Company	Catalog
**Alkaline Phosphatase**	AF647	1:100	B4-78	Human	BD	561500
**CD11c**	FITC	1:100	3.9	Human	BioLegend	301604
**CD104**	PE	1:100	58XB4	Human	BioLegend	327807
**CD146**	BV711	1:100	P1H12	Human	BD	563186
**CD19**	FITC	1:100	HIB19	Human	BioLegend	302206
**CD1a**	PE-Cy7	1:200	HI149	Human	BioLegend	300122
**CD205**	PE	1:400	HD30	Human	BioLegend	342203
**CD235ab**	Biotin	1:100	HIR2	Human	BioLegend	306618
**CD3**	Biotin	1:200	UCHT1	Human	BioLegend	300404
**CD3**	BV711	1:200	UCHT1	Human	BioLegend	300464
**CD31**	APC	1:100	WM59	human	BioLegend	303115
**CD33**	PE	1:25	WM53	Human	BD	555450
**CD33**	FITC	1:100	P67.6	Human	BioLegend	366619
**CD34**	BV605	1:100	581	Human	BioLegend	343530
**CD34**	APC	1:100	561	Human	BioLegend	343608
**CD4**	FITC	1:100	OKT4	Human	TONBO	35-0048
**CD4**	Biotin	1:100	SK3	Human	BioLegend	344610
**CD4**	BV605	1:100	OKT4	Human	BioLegend	317438
**CD45**	Biotin	1:100	a30-F11	Mouse	BioLegend	103104
**CD45**	PE	1:200	HI30	Human	BioLegend	304008
**CD45**	AF700	1:200	HI30	Human	BioLegend	304024
**CD45**	APC	1:200	HI30	Human	BioLegend	304011
**CD45**	Biotin	1:100	HI30	Human	BioLegend	304004
**CD45**	PE-Cy7	1:200	HI30	Human	BioLegend	304015
**CD49f**	AF488	1:200	GoH3	Human/Mouse	BioLegend	313608
**CD5**	PE	1:200	UCHT2	Human	BioLegend	300608
**CD56 (NCAM)**	FITC	1:100	HCD56	Human	BioLegend	318304
**CD69**	BV510	1:100	FN50	Human	BioLegend	310936
**CD7**	BV510	1:100	M-T701	Human	BD	563650
**CD8a**	APC	1:200	SK1	Human	TONBO	20-0087
**CD8a**	Biotin	1:200	RPA-T	Human	Biolegend	301004
**CD8b**	PE-Cy7	1:200	SIDI8BEE	Human	eBioscience	25-5273
**CD90**	AF700	1:200	5E10	Human	Biolegend	328119
**EpCAM (CD326)**	eFluor660	1:50	1B7	Human	eBioscience	50-9326
**EpCAM (CD326)**	PE-Cy7	1:100	9C4	Human	BioLegend	324222
**IFN-g**	PE-Cy7	1:50	4S.B3	Human	BioLegend	502527
**IL-2**	PE	1:50	MQ1-17H12	Human	BioLegend	500306
**NG2**	AF488	1:200	9.2.27	Human	BD	562413
**PDGFRa (CD140a)**	PE	1:50	16A1	Human	BioLegend	323506
**PDGFRb (CD140b)**	PE	1:100	18A2	Human	BioLegend	323606
**Streptavidin**	BV510	1:100	IP26	Human	BioLegend	405233
**TCR-ab**	AF700	1:100	IP26	Human	BioLegend	306730
**TNF-a**	APC	1:100	MAb11	Human	BioLegend	502812
**HLA-DR**	BV711	1:100	L243	human	BioLegend	307644

Supplementary Table 2 | List of Taqman qPCR probes (Integrated DNA Technologies)

Gene Name	RefSeq Number	IDT Assay Name
AIRE	NM_000383	Hs.PT.56a.2617893
CCL21	NM_002989	Hs.PT.58.26537771.g
CCL25	NM_005624	Hs.PT.58.1144750
CD205 (LY75)	NM_002349	Hs.PT.58.3240196
EPCAM	NM_002354	Hs.PT.58.39570895
FOXN1	NM_003593	Hs.PT.58.40389553
KITLG/SCF	NM_003994	Hs.PT.58.38688683
PAX1	NM_006192	Hs.PT.58.3033944
PAX9	NM_006194	Hs.PT.58.25853242
PSMB11/ b5T	NM_001099780	Hs.PT.58.40855890.g
TRP63	NM_001114981	Hs.PT.58.2966111
VIM	NM_003380	Hs.PT.58.38906895

Supplementary Table 3 | List of primary and secondary antibodies for immunostaining

Antigen	Host	Dilution	Company	Cat.No.
Aire	Rat	1:250	ThermoSci/ Invitrogen	14-9534-82
Caspase3	Rabbit	1:300	CellSignaling	9661
CD3-APC conjugated	Mouse	1:100	Biolegend	20-0038
CD3	Rabbit	1:100	Abcam	AB16669
CD11c-FITC conjuated	Mouse	1:100	Biolegend	301604
CD45-APC conjugated	Mouse	1:100	Biolegend	304011
CD45	Rabbit	1:100	Abcam	AB10558
CD49f- AF488 conjugated	Rat	1:100	Biolegend	313608
CD205-AF647 conjugated	Mouse	1:100	Biolegend	342206
CK5	Mouse	1:100	Abcam	AB17130
CK5/14	Rabbit	1:500	BioLegend	PRB-155P
CK8	Mouse	1:50	Abcam	AB9023
CK8	Guinea pig	1:100	Acris/2BeScientific	BP5075
CK8/18	Guinea pig	1:100	Acris/2BeScientific	BP5075
E-Cadherin	Rabbit	1:500	Abcam	AB40772
E-Cadherin	Mouse	1:100	BD	610181
Endomucin	Rat	1:100	SantaCruz	SC-65495
EpCAM	Rabbit	1:100	Sigma Aldrich	HPA026761
EpCAM- efluor660 cojugate	Mouse	1:100	eBioscience	50-9326-42
HLA-DR	Rabbit	1:200	Abcam	AB92511
Ki67	Rabbit	1:300	Chemicon	AB9260
p63	Mouse	1:50	Abcam	AB735
TE-7	Mouse	1:100	Merk	CBL271
Vimentin	Mouse	1:100	SantaCruz	SC-6260
Vimentin	Rabbit	1:100	CellSignaling	5741

Secondary Antibodies	Host		Company	Cat.No.
Anti-GP Cy3	Donkey	1:500	Jackson Immuno	706-165-148
Anti-Mouse AF488	Donkey	1:500	Jackson Immuno	715-545-150
Anti-Mouse Cy3	Donkey	1:500	Jackson Immuno	715-165-150
Anti-Mouse AF647	Donkey	1:500	Jackson Immuno	715-605-150
Anti-Rabbit AF488	Donkey	1:500	Jackson Immuno	711-545-152
Anti-Rabbit AF594	Donkey	1:500	Jackson Immuno	711-585-152
Anti-Rabbit AF647	Donkey	1:500	Jackson Immuno	711-605-152
Anti-Rat AF488	Donkey	1:500	Jackson Immuno	712-545-150
Anti-Rat AF 647	Donkey	1:500	Jackson Immuno	712-605-150
