# Supplementary material for: Reconstitution of a functional human thymus by postnatal stromal progenitor cells and natural whole-organ scaffolds
Source: Nat Commun. 2020 Dec 11;11:6372. doi: 10.1038/s41467-020-20082-7 (PMC7732825; doi:10.1038/s41467-020-20082-7)
Supplement: Supplementary file 2 — Description of Additional Supplementary Files [file 41467_2020_20082_MOESM2_ESM.pdf]

Description of Additional Supplementary Files

Supplementary Movie 1: Time-lapse recording of a growing clonal colony demonstrates high mobility of thymic epithelial cells (TEC). Image were acquired over a period of 4 days.

Supplementary Movie 2: 3D video rendering of a cannulated rat thymus generated from micro-CT data. A supero-inferior clipping plane has been applied to the data, this demonstrates differentiation of cortex and medullary structures following administration of I_2KI contrast prior to the scan. Video prepared using VG Studio MAX (Heidelberg, Germany).
