# Supplementary material for: Reconstitution of a functional human thymus by postnatal stromal progenitor cells and natural whole-organ scaffolds
Source: Nat Commun. 2020 Dec 11;11:6372. doi: 10.1038/s41467-020-20082-7 (PMC7732825; doi:10.1038/s41467-020-20082-7)
Supplement: Supplementary file 5 — Reporting Summary [file 41467_2020_20082_MOESM5_ESM.pdf]

nature research

Corresponding author(s): Paola Bonfanti

Last updated by author(s): 22/10/20

Reporting Summary

Nature Research wishes to improve the reproducibility of the work that we publish. This form provides structure for consistency and transparency in reporting. For further information on Nature Research policies, see Authors & Referees and the Editorial Policy Checklist .

Statistics

For all statistical analyses, confirm that the following items are present in the figure legend, table legend, main text, or Methods section.

n/a	Confirmed	
☐	☒	The exact sample size (*n*) for each experimental group/condition, given as a discrete number and unit of measurement
☐	☒	A statement on whether measurements were taken from distinct samples or whether the same sample was measured repeatedly
☐	☒	The statistical test(s) used AND whether they are one- or two-sided<br>*Only common tests should be described solely by name; describe more complex techniques in the Methods section.*
☒	☐	A description of all covariates tested
☐	☒	A description of any assumptions or corrections, such as tests of normality and adjustment for multiple comparisons
☐	☒	A full description of the statistical parameters including central tendency (e.g. means) or other basic estimates (e.g. regression coefficient) AND variation (e.g. standard deviation) or associated estimates of uncertainty (e.g. confidence intervals)
☐	☒	For null hypothesis testing, the test statistic (e.g. *F*, *t*, *r*) with confidence intervals, effect sizes, degrees of freedom and *P* value noted<br>*Give P values as exact values whenever suitable.*
☒	☐	For Bayesian analysis, information on the choice of priors and Markov chain Monte Carlo settings
☒	☐	For hierarchical and complex designs, identification of the appropriate level for tests and full reporting of outcomes
☒	☐	Estimates of effect sizes (e.g. Cohen's *d*, Pearson's *r*), indicating how they were calculated

Our web collection on statistics for biologists contains articles on many of the points above.

Software and code

Policy information about availability of computer code

Data collection	BD FACSDiva 8.01 software was used for flow cytometry data collection using FACSAria III machine or Fortessa X-20 machine, BD Bioscience. Applied Biosystems QuantStudio 3 Real-Time PCR software was used for qPCR data collection.<br>Zen 2.3 SP1 softwares were used for confocal microscope images acquisition.<br>R software with Seurat 3.1.1 package was used for single-cell dataset analysis on fresh human postnatal thymus<br>CellRanger Allignment and Read Counting software: CellRanger 3.0.2<br>EdgeR software package was used for examining differential expression of replicated count data in Bulk RNA-seq of cultured cells<br>The reference Genome is GRCh38
Data analysis	FlowJo 10.3 and 10.6.0, Volocity 6.3, Fiji 2.1.0, GraphPad Prism 8 and VG Studio MAX 2.2 were used for data analysis.

For manuscripts utilizing custom algorithms or software that are central to the research but not yet described in published literature, software must be made available to editors/reviewers. We strongly encourage code deposition in a community repository (e.g. GitHub). See the Nature Research guidelines for submitting code & software for further information.

Data

Policy information about availability of data

All manuscripts must include a data availability statement. This statement should provide the following information, where applicable:

- Accession codes, unique identifiers, or web links for publicly available datasets
- A list of figures that have associated raw data
- A description of any restrictions on data availability

RNA-sequencing data are deposited at ArrayExpress (https://www.ebi.ac.uk/arrayexpress/) under accession E-MTAB-9641;
scRNA-sequencing data are deposited at Gene Expression Omnibus (GEO) database (GEO, https://www.ncbi.nlm.nih.gov/geo/) under the accession code GSE159745

Field-specific reporting

Please select the one below that is the best fit for your research. If you are not sure, read the appropriate sections before making your selection.

[x] Life sciences [ ] Behavioural & social sciences [ ] Ecological, evolutionary & environmental sciences

For a reference copy of the document with all sections, see nature.com/documents/nr-reporting-summary-flat.pdf

Life sciences study design

All studies must disclose on these points even when the disclosure is negative.

Sample size	Sample size was determined based on preliminary data or published reports in the literature (Meran et al. 2020 Nature Medicine 2020; DOI: 10.1038/s41591-020-1024-z; Abarrategi, A. et al. J Clin Invest 127, 543-548, doi:10.1172/JCI89364 (2017); Bonfanti et al. Nature 2010 Aug 19;466(7309):978-82)
Data exclusions	Animals were excluded only if bone marrow was not reconstituted with human CD34+ cells in the in vivo grafting experiments with humanised mice.
Replication	Replicates for each experiment are specified in figure legend or text. All the experiments and results included in this study were confirmed with three or more independent experimental repeats. Statistical analysis was performed by using ANOVA unpaired unless stated otherwise.
Randomization	Littermates (females, nude and hairy) were randomly assigned for scaffold implantation, and a mouse code number allocated to each type of sample. Cells and scaffolds were injected or transplanted in a random order. The mouse ID number was then used to identify each sample. Corresponding ID and sample type was performed before the histology and/or FACS analysis
Blinding	Blinding was not possible because both control and repopulated scaffolds were assigned to independent mice; different analysis were conducted and repeated independently by more experimenters; to increase objectivity the samples were treated according to the mouse code number and not to the type of sample transplanted.

Reporting for specific materials, systems and methods

We require information from authors about some types of materials, experimental systems and methods used in many studies. Here, indicate whether each material, system or method listed is relevant to your study. If you are not sure if a list item applies to your research, read the appropriate section before selecting a response.

Materials & experimental systems

n/a	Involved in the study	
[ ]	[x]	Antibodies
[ ]	[x]	Eukaryotic cell lines
[x]	[ ]	Palaeontology
[ ]	[x]	Animals and other organisms
[ ]	[x]	Human research participants
[x]	[ ]	Clinical data

Methods

n/a	Involved in the study	
[x]	[ ]	ChIP-seq
[ ]	[x]	Flow cytometry
[x]	[ ]	MRI-based neuroimaging

Antibodies

Antibodies used	List of primary and secondary antibodies (including dilution) used for IHC and for FACS are provided in supplementary information (Supplementary Table 1 and 3):<br><br>Aire Rat 1:250 ThermoSci/ Invitrogen 14-9534-82

Caspase3 Rabbit 1:300 CellSignaling 9661
CD3-APC conjugated Mouse 1:100 Biolegend 20-0038
CD3 Rabbit 1:100 Abcam AB16669
CD11c-FITC conjuated Mouse 1:100 Biolegend 301604
CD45-APC conjugated Mouse 1:100 Biolegend 304011
CD45 Rabbit 1:100 Abcam AB10558
CD49f- AF488 conjugated Rat 1:100 Biolegend 313608
CD205-AF647 conjugated Mouse 1:100 Biolegend 342206
CK5 Mouse 1:100 Abcam AB17130
CK5/14 Rabbit 1:500 BioLegend PRB-155P
CK8 Mouse 1:50 Abcam AB9023
CK8 Guinea pig 1:100 Acris/2BeScientific BP5075
CK8/18 Guinea pig 1:100 Acris/2BeScientific BP5075
E-Cadherin Rabbit 1:500 Abcam AB40772
E-Cadherin Mouse 1:100 BD 610181
Endomucin Rat 1:100 SantaCruz SC-65495
EpCAM Rabbit 1:100 Sigma Aldrich HPA026761
EpCAM- efluor660 cojugated Mouse 1:100 eBioscience 50-9326-42
HLA-DR Rabbit 1:200 Abcam AB92511
Ki67 Rabbit 1:300 Chemicon AB9260
p63 Mouse 1:50 Abcam AB735
TE-7 Mouse 1:100 Merk CBL271
Vimentin Mouse 1:100 SantaCruz SC-6260
Vimentin Rabbit 1:100 CellSignaling 5741

Alkaline Phosphatase AF647 1:100 B4-78 Human BD 561500
CD11c FITC 1:100 3.9 Human BioLegend 301604
CD104 PE 1:100 58XB4 Human BioLegend 327807
CD146 BV711 1:100 P1H12 Human BD 563186
CD19 FITC 1:100 HIB19 Human BioLegend 302206
CD1a PE-Cy7 1:200 HI149 Human BioLegend 300122
CD205 PE 1:400 HD30 Human BioLegend 342203
CD235ab Biotin 1:100 HIR2 Human BioLegend 306618
CD3 Biotin 1:200 UCHT1 Human BioLegend 300404
CD3 BV711 1:200 UCHT1 Human BioLegend 300464
CD31 APC 1:100 WM59 human BioLegend 303115
CD33 PE 1:25 WM53 Human BD 555450
CD33 FITC 1:100 P67.6 Human BioLegend 366619
CD34 BV605 1:100 581 Human BioLegend 343530
CD34 APC 1:100 561 Human BioLegend 343608
CD4 FITC 1:100 OKT4 Human TONBO 35-0048
CD4 Biotin 1:100 SK3 Human BioLegend 344610
CD4 BV605 1:100 OKT4 Human BioLegend 317438
CD45 Biotin 1:100 a30-F11 Mouse BioLegend 103104
CD45 PE 1:200 HI30 Human BioLegend 304008
CD45 AF700 1:200 HI30 Human BioLegend 304024
CD45 APC 1:200 HI30 Human BioLegend 304011
CD45 Biotin 1:100 HI30 Human BioLegend 304004
CD45 PE-Cy7 1:200 HI30 Human BioLegend 304015
CD49f AF488 1:200 GoH3 Human/Mouse BioLegend 313608
CD5 PE 1:200 UCHT2 Human BioLegend 300608
CD56 (NCAM) FITC 1:100 HCD56 Human BioLegend 318304
CD69 BV510 1:100 FN50 Human BioLegend 310936
CD7 BV510 1:100 M-T701 Human BD 563650
CD8a APC 1:200 SK1 Human TONBO 20-0087
CD8a Biotin 1:200 RPA-T Human Biolegend 301004
CD8b PE-Cy7 1:200 SIDI8BEE Human eBioscience 25-5273
CD90 AF700 1:200 5E10 Human Biolegend 328119
EpCAM (CD326) eFluor660 1:50 1B7 Human eBioscience 50-9326
EpCAM (CD326) PE-Cy7 1:100 9C4 Human BioLegend 324222
IFN-g PE-Cy7 1:50 4S.B3 Human BioLegend 502527
IL-2 PE 1:50 MQ1-17H12 Human BioLegend 500306
NG2 AF488 1:200 9.2.27 Human BD 562413
PDGFRa (CD140a) PE 1:50 16A1 Human BioLegend 323506
PDGFRb (CD140b) PE 1:100 18A2 Human BioLegend 323606

Streptavidin BV510 1:100 IP26 Human BioLegend 405233
TCR-ab AF700 1:100 IP26 Human BioLegend 306730
TNF-a APC 1:100 MAb11 Human BioLegend 502812
HLA-DR BV711 1:100 L243 human BioLegend 307644
Feeder PE 1:100 RMV-7 Mouse Miltenyi 130120166

Validation

- FACS antibodies purchased from Biolegends have been validated according to manufacturer's instructions: https://www.biolegend.com/nl-nl/reproducibility

- FACS antibodies purchased from EBIOSCIENCE:
CD8b PE-Cy7 (SIDI8BEE, Human, eBioscience)
Staining of normal human peripheral blood cells with Anti-Human CD8a FITC (Product # 11-0087-42) and Mouse IgG1 K Isotype Control PE-Cyanine7 (Product # 25-4714-80) (left) or Anti-Human/Non-Human Primate CD8b PE-Cyanine7 (right). Cells in the lymphocyte gate were used for analysis.
https://www.thermofisher.com/antibody/product/CD8b-Antibody-clone-SIDI8BEE-Monoclonal/25-5273-42

CD31 biotin (WM-59, Human, eBioscience)
Surface staining of normal human peripheral blood cells with Anti-Human CD31 (PECAM-1) FITC (left), and PE (right). Appropriate isotype controls were used (open histogram). Cells in the monocyte population were used for analysis.
https://www.thermofisher.com/antibody/product/CD31-PECAM-1-Antibody-clone-WM-59-WM59-Monoclonal/13-0319-82

- FACS antibodies purchased from BD:
AP, References: https://www.bdbiosciences.com/eu/applications/research/stem-cell-research/pluripotent-stem-cell-markers-esc-and-ipsc/human/alexa-fluor-647-mouse-anti-human-alkaline-phosphatase-b4-78/p/561500

CD146, references:
https://www.bdbiosciences.com/us/applications/research/stem-cell-research/mesenchymal-stem-cell-markers-adipose/human/positive-markers/bv711-mouse-anti-human-cd146-p1h12/p/563186

CD33 PE, references:
https://www.bdbiosciences.com/eu/applications/research/stem-cell-research/hematopoietic-stem-cell-markers/human/negative-markers/pe-mouse-anti-human-cd33-wm53-also-known-as-wm-53/p/555450

CD7 BV510, references:
https://www.bdbiosciences.com/us/reagents/research/antibodies-buffers/immunology-reagents/anti-human-antibodies/cell-surface-antigens/bv510-mouse-anti-human-cd7-m-t701/p/563650

NG2 AF488, references:
https://www.bdbiosciences.com/eu/reagents/research/antibodies-buffers/cell-biology-reagents/cell-biology-antibodies/alexa-fluor-488-mouse-anti-chondroitin-sulfate-9227/p/562413

Primary antibody validation:
Aire Pubmed ID: 1500592
Caspase3 Pubmed ID: 29176575
CD3, Pubmed ID: 18658050
CD45, Validated by Manufacturers
CK5, Pubmed ID: 17065488
CK5/14, Pubmed ID: 23736260
CK8, Validated by Manufacturers
CK8/18, Validated by Manufacturers
E-Cadherin, Pubmed ID: 24915897
E-Cadherin, Pubmed ID: 10528188
Endomucin, Pubmed ID: 18924607
EpCAM, Validated by HPA
HLA-DR, Validated by Manufacturers
Ki67, Validated by Manufacturers
p63, Pubmed ID: 15800938
TE-7, Pubmed ID: 25650991
Vimentin, Pubmed ID: 11111111
Vimentin, Pubmed ID: 15766329

Eukaryotic cell lines

Policy information about cell lines

Cell line source(s)	HuVEC-VeraVec cell line were from Angiocrine, CAT: HVERA101; 3T3-J2 cells were originally developed in Howard Green laboratory in Harvard, and kindly provided by Yann Barrandon. References: Rheinwald and Green, 1975. Cell; Barrandon et Green, PNAS, 1987; Gallico et al., NEJM, 1984.
Authentication	Cell lines were authenticated by STR profiling (for human lines) and species identification for validation.
Mycoplasma contamination	The cell lines used in this study was tested for Mycoplasma contamination and resulted negative.
Commonly misidentified lines (See ICLAC register)	No commonly misidentified cell lines were used in this study

Animals and other organisms

Policy information about studies involving animals; ARRIVE guidelines recommended for reporting animal research

Laboratory animals	Mice: females 8-14 week old.<br>- NOD.Prkdcscid Il2rgtm1Wjl/J. The Jackson Laboratory (abarrategi<br>- NOD.Cg-Foxn1em1Dvs Prkdcscid Il2rgtm1Wjl/J. The Jackson Laboratory, Stock No: 026263<br>Mice were maintained on a 12 h light–dark cycle, ambient temperature 19/22°C, and humidity 45/65%<br><br>Rats: males 150-200gr (6-8 week old)<br>- Sprague Dawley rats. Charles River
Wild animals	No wild animals were used in the study
Field-collected samples	No field-collected samples were used in the study
Ethics oversight	All animal procedures were in accordance with ethical approval and UK Home Office Project License (PPL) 70/8904 and PDD3A088A and approved by the Crick and UCL Institutes's Animal Welfare and Ethical Review Body (UK).

Note that full information on the approval of the study protocol must also be provided in the manuscript.

Human research participants

Policy information about studies involving human research participants

Population characteristics	Postnatal thymi were donated by patients (age range 3 days- 11 years old) undergoing cardiothoracic surgery at the Great Ormond Street Hospital
Recruitment	A written informed consent was obtained from the patient parents or legally authorised representatives
Ethics oversight	This study was approved by the NRES Committee Yorkshire & The Humber - South Yorkshire 15-YH-0334.

Note that full information on the approval of the study protocol must also be provided in the manuscript.

Flow Cytometry

Plots

Confirm that:

- [x] The axis labels state the marker and fluorochrome used (e.g. CD4-FITC).
- [x] The axis scales are clearly visible. Include numbers along axes only for bottom left plot of group (a 'group' is an analysis of identical markers).
- [x] All plots are contour plots with outliers or pseudocolor plots.
- [x] A numerical value for number of cells or percentage (with statistics) is provided.

Methodology

Sample preparation	Cell suspension preparation was dependent on sample type (i.e. trypsinisation for cells in culture, enzymatic and mechanical dissociation for human or murine tissue). Tissue preparation is therefore specified in appropriate sections of Material and Methods.
Instrument	FACSAria III machine or Fortessa X-2 machine, BD Bioscience.

Software	BD FACSDiva software and FlowJoTM.
Cell population abundance	Cell population abundance is specified in main text and figure legend, accordingly to the relative experiment.<br>mTECType1: 6035 sorted events (72% viable); mTECType2: 54446 (96% viable); cTECType1: 8781 (74% viable); cTECType2 23379 (96%). Triple negative: 500K to 4 millions sorted events. CD34 500K to 1 million sorted events.<br>Purity check was assessed soon after sorting by running 10 ul of sorted sample into 100 ul of FACS buffer.
Gating strategy	Cells were first gated based on light scatter properties (FSC-A) and granularity (SSC-A), and doublets were excluded by gating SSC Width against Hight. Dead cells were excluded on the basis of their positivity for Live/Dead (L/D) markers (Zombie, Biolegend or DAPI, Sigma). Cells expressing relevant markers were classified based on unstained negative cells or L/D only staining control.

☒ Tick this box to confirm that a figure exemplifying the gating strategy is provided in the Supplementary Information.
